# Supplementary material for: A systematic review of neurological impairments in myalgic encephalomyelitis/ chronic fatigue syndrome using neuroimaging techniques
Source: PLoS One. 2020 Apr 30;15(4):e0232475. doi: 10.1371/journal.pone.0232475 (PMC7192498; doi:10.1371/journal.pone.0232475)
Supplement: S2 File — (DOCX) [file pone.0232475.s002.docx]

**S2. Extended search term code.**

| Database | Code |
| --- | --- |
| PUBMED | ("Fatigue Syndrome, Chronic"[Mesh]) AND (("Functional Neuroimaging"[Mesh]) OR ("Neuroimaging"[Mesh]) OR ("Diagnostic Imaging"[Mesh]) OR ("Tomography, X-Ray Computed"[Mesh]) OR ("Magnetic Resonance Imaging"[Mesh]) OR ("Magnetic Resonance Spectroscopy"[Mesh]) OR ("Magnetoencephalography"[Mesh]) OR ("Positron-Emission Tomography"[Mesh]) OR ("Tomography, Emission-Computed, Single-Photon"[Mesh]) OR ("Electroencephalography"[Mesh])) |
| EMBASE  SCOPUS  ProQuest | (( "Chronic Fatigue Syndrome") OR ( "Myalgic Encephalomyelitis") OR (“Encephalomyelitis, Myalgic”) OR (“Chronic Fatigue Syndromes”) OR (“Fatigue Syndromes, Chronic”) OR (“Chronic Fatigue-Fibromyalgia Syndrome”) OR (“Chronic Fatigue Fibromyalgia Syndrome”) OR (“Chronic Fatigue Fibromyalgia Syndromes”) OR (“Fatigue-Fibromyalgia Syndrome, Chronic”) OR (“Fatigue-Fibromyalgia Syndromes, Chronic”) OR (“Postviral Fatigue Syndrome”) OR (“Infectious Mononucleosis-Like Syndrome, Chronic”) OR (“Infectious Mononucleosis Like Syndrome, Chronic”) OR (“Royal Free Disease”) OR (“Chronic Fatigue and Immune Dysfunction Syndrome”) OR (“Chronic Fatigue Disorder”) OR (“Chronic Fatigue Disorders”) OR (“Fatigue Disorder, Chronic”) OR (“Fatigue Disorders, Chronic”) OR ( "systemic exertion intolerance" ) OR (“Fatigue Syndrome, Postviral”) OR (“Fatigue Syndromes, Postviral”) OR (“Postviral Fatigue Syndromes”)) AND ((“Functional Neuroimaging”) OR (“Neuroimaging, Functional”) OR (“Functional Brain Imaging”) OR (“Brain Imaging, Functional”) OR (“Brain Imagings, Functional”) OR (“Functional Brain Imagings”) OR (“Imaging, Functional Brain”) OR (“Imagings, Functional Brain”) OR (“Neuroimaging”) OR (“Brain Imaging”) OR (“Imaging, Brain”) OR (“diagnostic imaging”) OR (“Imaging, Diagnostic”) OR (“Medical Imaging”) OR (“Imaging, Medical”) OR (“X-Ray Computed Tomography”) OR (“Tomography, X-Ray Computerized”) OR (“Tomography, X Ray Computerized”) OR (“Computed X Ray Tomography”) OR (“X-Ray Computer Assisted Tomography”) OR (“X Ray Computer Assisted Tomography”) OR (“Tomography, X-Ray Computer Assisted”) OR (“Tomography, X Ray Computer Assisted”) OR (“Computerized Tomography, X Ray”) OR (“Computerized Tomography, X-Ray”) OR (“X-Ray Computerized Tomography”) OR (“CT X Ray”) OR (“CT X Rays”) OR (“X Ray, CT”) OR (“X Rays, CT”) OR (“Tomodensitometry”) OR (“Tomography, X Ray Computed”) OR (“X Ray Tomography, Computed”) OR (“X-Ray Tomography, Computed”) OR (“Computed X-Ray Tomography”) OR (“Tomographies, Computed X-Ray”) OR (“Tomography, Computed X-Ray”) OR (“Tomography, Xray Computed”) OR (“Computed Tomography, Xray”) OR (“Xray Computed Tomography”) OR (“CAT Scan, X Ray”) OR (“CAT Scan, X-Ray”) OR (“CAT Scans, X-Ray”) OR (“Scan, X-Ray CAT”) OR (“Scans, X-Ray CAT”) OR (“X-Ray CAT Scan”) OR (“X-Ray CAT Scans”) OR (“Tomography, Transmission Computed”) OR (“Computed Tomography, Transmission”) OR (“Transmission Computed Tomography”) OR (“CT Scan, X-Ray”) OR (“CT Scan, X Ray”) OR (“CT Scans, X-Ray”) OR (“Scan, X-Ray CT”) OR (“Scans, X-Ray CT”) OR (“X-Ray CT Scan”) OR (“X-Ray CT Scans”) OR (“Computed Tomography, X-Ray”) OR (“Computed Tomography, X Ray”) OR (“X Ray Computerized Tomography”) OR (“Cine-CT”) OR (“Cine CT”) OR (“Electron Beam Computed Tomography”) OR (“Electron Beam Tomography”) OR (“Beam Tomography, Electron”) OR (“Tomography, Electron Beam”) OR (“Tomography, X-Ray Computerized Axial”) OR (“Tomography, X Ray Computerized Axial”) OR (“X-Ray Computerized Axial Tomography”) OR (“X Ray Computerized Axial Tomography”) OR (“Imaging, Magnetic Resonance”) OR (“NMR Imaging”) OR (“Imaging, NMR”) OR (“Tomography, NMR”) OR (“Tomography, MR”) OR (“MR Tomography”) OR (“NMR Tomography”) OR (“Steady-State Free Precession MRI”) OR (“Steady State Free Precession MRI”) OR (“Zeugmatography”) OR (“Imaging, Chemical Shift”) OR (“Chemical Shift Imagings”) OR (“Imagings, Chemical Shift”) OR (“Shift Imaging, Chemical”) OR (“Shift Imagings, Chemical”) OR (“Chemical Shift Imaging”) OR (“Tomography, Proton Spin”) OR (“Proton Spin Tomography”) OR (“Magnetization Transfer Contrast Imaging”) OR (“MRI Scans”) OR (“MRI Scan”) OR (“Scan, MRI”) OR (“Scans, MRI”) OR (“fMRI”) OR (“MRI, Functional”) OR (“Functional MRI”) OR (“Functional MRIs”) OR (“MRIs, Functional”) OR (“Functional Magnetic Resonance Imaging”) OR (“Magnetic Resonance Imaging, Functional”) OR (“Spin Echo Imaging”) OR (“Echo Imaging, Spin”) OR (“Echo Imagings, Spin”) OR (“Imaging, Spin Echo”) OR (“Imagings, Spin Echo”) OR (“Spin Echo Imagings”) OR (“Magnetic Resonance Spectroscopies”) OR (“Resonance Spectroscopy, Magnetic”) OR (“MR Spectroscopy”) OR (“Spectroscopy, MR”) OR (“Nuclear Magnetic Resonance”) OR (“Magnetic Resonance, Nuclear”) OR (“Resonance, Nuclear Magnetic”) OR (“In Vivo NMR Spectroscopy”) OR (“NMR Spectroscopy, In Vivo”) OR (“Magnetic Resonance”) OR (“Resonance, Magnetic”) OR (“Spectroscopy, Nuclear Magnetic Resonance”) OR (“Spectroscopy, NMR”) OR (“NMR Spectroscopy”) OR (“NMR Spectroscopies”) OR (“Spectroscopies, NMR”) OR (“Positron Emission Tomography”) OR (“PET Scan”) OR (“PET Scans”) OR (“Scan, PET”) OR (“Scans, PET”) OR (“Tomography, Positron-Emission”) OR (“Tomography, Positron Emission”) OR (“Tomography, Emission-Computed, Single-Photon”) OR (“CT Scan, Single-Photon Emission”) OR (“CT Scan, Single Photon Emission”) OR (“Radionuclide Tomography, Single-Photon Emission-Computed”) OR (“Radionuclide Tomography, Single Photon Emission Computed”) OR (“Tomography, Single-Photon, Emission-Computed”) OR (“Single-Photon Emission Computerized Tomography”) OR (“Single Photon Emission Computerized Tomography”) OR (“Single-Photon Emission CT Scan”) OR (“Single Photon Emission CT Scan”) OR (“Single-Photon Emission-Computed Tomography”) OR (“Emission-Computed Tomography, Single-Photon”) OR (“Single Photon Emission Computed Tomography”) OR (“Tomography, Single-Photon Emission-Computed”) OR (“SPECT”) OR (“CAT Scan, Single-Photon Emission”) OR (“CAT Scan, Single Photon Emission”) OR (“Single-Photon Emission Computer-Assisted Tomography”) OR (“Single Photon Emission Computer Assisted Tomography”) OR (“Electroencephalography”) OR (“EEG”) OR (“Electroencephalogram”) OR (“Electroencephalograms”)) |
