# Supplementary material for: A systematic review of neurological impairments in myalgic encephalomyelitis/ chronic fatigue syndrome using neuroimaging techniques
Source: PLoS One. 2020 Apr 30;15(4):e0232475. doi: 10.1371/journal.pone.0232475 (PMC7192498; doi:10.1371/journal.pone.0232475)
Supplement: S3 File — (DOCX) [file pone.0232475.s003.docx]

**S3.** Summary of study characteristics table

|  | **Year** | **Study Design** | **Dx** | **Sample sizes** |  | **Method of analysis** |
| --- | --- | --- | --- | --- | --- | --- |
|  |  |  |  | **ME/CFS** | **Control** |  |
| Armitage et al. | 2009 | Observational Twin Study | Fukuda (1994) | 13 | 13 | EEG/PSG |
| Barnden et al. | 2011 | Observational Case- control | Fukuda (1994) Canadian Criteria (2003) | 25 | 25 | T1w and T2w 1.5T MRI |
| Barnden et al. | 2015 | Observational Case- control | Fukuda (1994) Canadian Criteria (2003) | 25 | 25 | T1w and T2w 1.5T MRI |
| Barnden et al. | 2016 | Observational Case- control | Fukuda (1994) | 25 | 25 | T1w and T2w 1.5T MRI |
| Barnden et al. | 2018 | Observational  Case-control | Canadian Criteria (2003) | 43 | 27 | T1w and T2w 1.5T MRI |
| Biswal et al. | 2011 | Observational Case-control | Fukuda (1994) | 11 | 10 | ASL 3T fMRI |
| Boissoneault et al. | 2016 | Observational Case-Control | Fukuda (1994) | 17 | 17 | ASL 3T fMRI |
| Boissoneault et al. | 2018 | Observational Case-Control | Fukuda (1994) | 19 | 15 | ASL 3T fMRI |
| Boissoneault et al. | 2019 | Observational Case-control | Fukuda (1994) | 14 | 14 | ASL 3T fMRI |
| Caseras et al. | 2006 | Observational Case-Control | Fukuda (1994) | 18 | 12 | T2w 1.5T fMRI |
| Caseras et al. | 2008 | Observational Case-Control | Fukuda (1994) | 13 | 12 | T2w BOLD 1.5T fMRI |
| Chaudhuri et al. | 2002 | Observational Case-Control | Fukuda (1994) | 8 | 8 | 1.5T ^1^H MRS |
| Cleare et al. | 2005 | Observational Case-control | Fukuda (1994) | 10 | 10 | PET and [11C]WAY-100635 |
| Cook et al. | 2007 | Observational Case-control | Fukuda (1994) | 9 | 11 | BOLD 3T fMRI |
| de Lange et al. | 2004 | Observational Case-control | Fukuda (1994) | 16 | 16 | Rapid event- related 1.5T fMRI |
| de Lange et al. | 2005 | Observational Case-control | Fukuda (1994) | 13 | 13 | VBM 1.5T MRI |
| Decker et al. | 2009 | Observational Case-control | Fukuda (1994) | 35 | 40 | EEG/ PSG |
| Finkelmeyer et al | 2018A | Observational Case-control | Fukuda (1994) | 42 | 30 | VBM 3T MRI |
| Finkelmeyer et al | 2018B | Observational Case-control | Fukuda (1994) | 40 | 10 | phase-contrast, 3T qfMRI |
| Flor-Henry et al | 2010 | Observational Case-control | Fukuda (1994) | 61 | 80 | EEG |
| Gay et al | 2016 | Observational Case-control | Fukuda (1994) Canadian Criteria (2003) | 19 | 17 | 3T MRI |
| Kim et al | 2015 | Observational Case-control | Fukuda (1994) | 18 | 18 | 3T fMRI |
| Lange et al. | 2005 | Observational Case-control | Fukuda (1994) | Study 1: 172  Study 2: 290 | Study 1: 100  Study 2: 126 | BOLD 1.5T fMRI |
| Le Bon et al | 2012 | Observational Case-control | Fukuda (1994) | 10 | 10 | EEG |
| Lewis et al. | 2001 | Observational Twin Study | Fukuda (1994) | 22 | 22 | fMRI SPECT |
| Mathew et al. | 2008 | Observational Case-control | Fukuda (1994) | 16 | 15 | T1w MRI  3T ^1^H MRS |
| Miller et al. | 2014 | Observational Case-control | Fukuda (1994) | 71 | 212 | T2w 3T fMRI |
| Mueller et al. | 2019 | Observational Case-control | Fukuda (1994) | 15 | 15 | 3T MRS - whole-brain echo-planar spectroscopic imaging |
| Murrough et al. | 2010 | Observational Case-control | Fukuda (1994) | 17 | 19 | ^1^H MRS |
| Nakatomi et al. | 2014 | Observational Case-control | Fukuda (1994) | 9 | 10 | C-(R)-PK11195 PET |
| Neu et al | 2011 | Observational Case-control | Fukuda (1994) | 15 | 16 | EEG P300 |
| Neu et al | 2014 | Observational Case-control | Fukuda (1994) | 52 | 25 | EEG spectral analysis |
| Okada et al | 2004 | Observational Case-control | Fukuda (1994) | 16 | 49 | VBM 3T MRI |
| Puri et al | 2002 | Observational Case-control | Fukuda (1994) | 8 | 8 | 1.5T ^1^H MRS |
| Puri et al | 2012 | Observational Case-control | Fukuda (1994) | 26 | 26 | VBM 3T MRI study |
| Schmaling et al. | 2003 | Observational Case-control | Fukuda (1994) | 15 | 15 | fMRI SPECT |
| Shan et al. | 2016 | Observational Case-control | Fukuda (1994) Canadian Criteria (2003) | 15 | 10 | Longitudinal 1.5T MRI |
| Shan et al. | 2017 | Observational Case-control | Canadian Criteria (2003) | 38 | 14 | T1w and  T2w 1.5T MRI |
| Shan et al. | 2018A | Observational Case-control | Fukuda (1994) | 45 | 27 | BOLD 3T fMRI |
| Shan et al. | 2018B | Observational Case-control | Fukuda (1994) | 43 | 26 | tfMRI |
| Sherlin et al. | 2007 | Observational Twin Study | Fukuda (1994) | 17 | 17 | EEG LORETA |
| Shungu et al. | 2012 | Observational Case-control | Fukuda (1994) | 15 | 13 | AS fMRI  3T MRS |
| Siessmeier et al. | 2003 | Observational Case-control | Fukuda (1994) | 26 | 18 | FDG-PET |
| Staud et al | 2018 | Observational Case-control | Fukuda (1994) Canadian Criteria (2003) | 17 | 16 | ASL 3T fMRI |
| Tanaka et al | 2006 | Observational Case-control | Fukuda (1994) | 6M | 7M | 3T fMRI |
| van der Schaaf et al. | 2017 | Observational Case-control | Fukuda (1994) | 89 | 26 | 3T MRI |
| van der Schaaf et al. | 2018 | Observational Case-control | Fukuda (1994) | 94 | 30 | MRI |
| Vuong et al. | 2019 | Observational Case-control | Fukuda (1994) | CFS (TMD+): 16  CFS (TMD-): 26 | 10 | T1w 3T MRI |
| Wu et al. | 2016 | Observational Case-control | Fukuda (1994) | 24 | 23 | EEG |
| Yamamoto et al. | 2004 | Observational Case-control | Fukuda (1994) | 10 | 10 | 0.3T MRI  PET |
| Yamamoto et al. | 2012 | Observational Case-control | Fukuda (1994) | CFS (-): 6 CFS (+): 5 | 11 | PET |
| Zeinah et al. | 2015 | Observational Case-control | Fukuda (1994) | 15 | 14 | T1w, DTI, ASL 3T  MRI |
| Zinn et al. | 2016 | Observational Case-control | Canadian Criteria (2003) | 9 | 9 | eLORETA EEG |
| Zinn et al | 2017 | Observational Case-control | Fukuda (1994) | 14 | 15 | qEEG |
| Zinn et al | 2018 | Observational Case-control | Fukuda (1994) | 50 | 50 | eLORETA qEEG |

ASL, arterial spin labelling; BOLD, blood oxygenation level dependent; ME/CFS, myalgic encephalomyelitis/ chronic fatigue syndrome; DX, diagnostic criteria; EEG, electroencephalogram; eLORETA, exact low-resolution brain electromagnetic tomography; fMRI, functional magnetic resonance imaging; MRI, magnetic resonance imaging; MRS, magnetic resonance spectroscopy; PSG, polysomnography; PET, positron emission tomography; qfMRI; quantitative functional magnetic resonance imaging; SPECT, single-photon emission computed tomography; T1W, T1-weighted; T2W- weighted; VBM, voxel-based morphometry
