# Supplementary material for: A systematic review of neurological impairments in myalgic encephalomyelitis/ chronic fatigue syndrome using neuroimaging techniques
Source: PLoS One. 2020 Apr 30;15(4):e0232475. doi: 10.1371/journal.pone.0232475 (PMC7192498; doi:10.1371/journal.pone.0232475)
Supplement: S4 File — (DOCX) [file pone.0232475.s004.docx]

**S4.** Summary of participant characteristics table

| Reference | Dx | Sample (n) |  | Age (years, mean (SD) |  | Sex, female (%) |  | Illness duration (years) | BMI (kg m−2), mean (SD) |  | Weight (kg), mean (SD) |  | % right-handed |  |
| --- | --- | --- | --- | --- | --- | --- | --- | --- | --- | --- | --- | --- | --- | --- |
|  |  | CFS | HC | CFS | HC | CFS | HC |  | CFS | HC | CFS | HC | CFS | HC |
| Armitage *et al*. (2009) | Fukuda (1994) | 13 | 13 | 45.4 (10.0) | 45.4 (10.0) | 100% | 100% | 9.2 | 30.1 (7.2) | 29.7 (6.1) | NR | NR | NR | NR |
| Barnden *et al*. (2011) | Fukuda (1994) Canadian Criteria (2011) | 25 | 25 | 31.7 (8.8) | 33.7 (10.3) | 76% | 76% | NR | NR | NR | 67.6 (10.9) | 68.9 (14.0) | NR | NR |
| Barnden *et al*. (2015) | Canadian Criteria (2011) | 25 | 25 | 32 (19-46) | 32.8 (20-46) | 76% | 76% | 7.4 (range, 2-15) | NR | NR | NR | NR | NR | NR |
| Barnden *et al*. (2016) | Canadian criteria (2011) | 25 | 25 | 32 (19-46) | 32.8 (20-46) | 76% | 76% | 7.4 (range, 2-15) | NR | NR | NR | NR | NR | NR |
| Barnden *et al*. (2018) | Fukuda (1994) | 43 | 27 | NR | NR | NR | NR | NR | NR | NR | NR | NR | NR | NR |
| Biswal *et al*. (2011) | Fukuda (1994) | 11 | 10 | NR | NR | NR | NR | NR | NR | NR | NR | NR | NR | NR |
| Boissoneault *et al*. (2016) | Fukuda (1994) | 17 | 17 | 48.88 (12) | 49.82 (11.32) | 100% | 100% | 11.78 (9.12) | NR | NR | NR | NR | NR | NR |
| Boissoneault *et al*. (2018) | Fukuda (1994) | 14 | 14 | 48.57 (12.11) | 49.57 (13.16) | 100% | 100% | 12.81 (9.62) | NR | NR | NR | NR | NR | NR |
| Boissoneault *et al*. (2019) | Fukuda (1994) | 17 | 17 | 48.57 (12.11) | 49.57 (13.16) | 100% | 100% | 12.81 (9.62) | NR | NR | NR | NR | NR | NR |
| Caseras *et al*. (2006) | Fukuda (1994) | 17 | 12 | 33.53 (6.17) | 33.50 (7.12) | 58.8% | 66.7% | NR | NR | NR | NR | NR | 100% | 100% |
| Caseras *et al*. (2008) | Fukuda (1994) | 13 | 12 | 33.75 (7.64) | 34.36 (6.77) | 61.5% | 63.6% | NR | NR | NR | NR | NR | 100% | 100% |
| Chaudhuri et al. (2002) | Fukuda (1994) | 8 | 8 | 32-54 | 28-59 | 87.5% | NR | 2-14 | NR | NR | NR | NR | NR | NR |
| Cleare *et al*. (2005) | Fukuda (1994) | 10 | 10 | 46.5 (5.9) | 40.7 (10.0) | 20% | 10% | 3.1 (2.0) | NR | NR | 82.0 (14.4) | 86.1 (10) | 100% | 100% |
| Cook *et al*. (2007) | Fukuda (1994) | 9 | 11 | 43 (8.0) | 42 (9.0) | 66.7% | 72.7% | NR | NR | NR | 77.11 (18.6) | 67.13 (13.6) | NR | NR |
| de Lange *et al*. (2004) | Fukuda (1994) | 16 | 16 | 28.4 (6.0) | 24.9 (6.4) | 100% | 100% | 6.3 (4.4) | NR | NR | NR | NR | 100% | 100% |
| de Lange *et al*. (2005) | Fukuda (1994) | Cohort 1: 13  Cohort 2: 15 | Cohort 1: 15  Cohort 2: 13 | Cohort 1: 28.9 (6.1)  Cohort 2: 43.9 (14.4) | Cohort 1: 25.7 (6.5)  Cohort 2: 43.4 (14.1) | Cohort 1: 100%  Cohort 2: 100% | Cohort 1: 100%  Cohort 2: 100% | NR | NR | NR | NR | NR | NR | NR |
| Decker *et al*. (2009) | Fukuda (1994) | 35 | 40 | Males: 47.0 (0.7)  Females: 50.9 (9.1) | Males: 48.0 (10.9)  Females: 50.8 (8.1) | 85.7% | 90% | NR | Males: 28.2 (5.4)  Females: 28.8 (4.1) | Males: 29.5 (5.7)  Females: 29.4 (8.1) | NR | NR | NR | NR |
| Finkelmeyer *et al*. (2018A) | Fukuda (1994) | 40 | 10 | 45.3 (11.6) | 49.4 (15.3) | 75% | 70% | 13.0 (9.31) | 26.0 (5.1) | 26.7 (4.6) | NR | NR | NR | NR |
| Finkelmeyer *et al*. (2018B) | Fukuda (1994) | 42 | 28 | 45.6 (11.7) | 48.4 (11.3) | 76% | 67.9% | NR | NR | NR | NR | NR | NR | NR |
| Flor-Henry *et al.* (2010) | Fukuda (1994) | 61 | 80 | 45.65 (9.9) | 25.57 (8.37) | 100% | 100% | NR | NR | NR | NR | NR | 100% | 100% |
| Gay *et al.* (2016) | Fukuda (1994) | 19 | 17 | 52.33 (10.63) | 48.75 (11.75) | 100% | 100% | NR | NR | NR | NR | NR | NR | NR |
| Kim *et al*. (2015) | Fukuda (1994) | 18 | 18 | 43.9 (4.8) | 45.9 (3.2) | 100% | 100% | NR | NR | NR | NR | NR | 100% | 100% |
| Lange *et al.* (2005) | Fukuda (1994) | Study 1: 6  Study 2: 19 | Study 1: 7  Study 2: 15 | Study 1: 38.17 (9.0)  Study 2: 37.53 (8.0) | Study 1: 30.71 (9.6)  Study 2: 30.80 (7.5) | Study 1: 100%  Study 2: 84% | Study 1: 57%  Study 2: 68% | NR | NR | NR | NR | NR | 100% | 100% |
| Le Bon *et al.* (2012) | Fukuda (1994) | 10 | 10 | 30.7 (8.0) | 32.2 (9.8) | 100% | 100% | NR | 22.9 (4.3) | 21.5 (1.9) | NR | NR | NR | NR |
| Lewis *et al*. (2001) | Fukuda (1994) | 22 | 22 | 41.4 (19-57) | 41.4 (19-57) | 90.9% | 90.9% | 7.0 | NR | NR | NR | NR | NR | NR |
| Mathew *et al.* (2008) | Fukuda (1994) | 16 | 15 | 37.6 (9.9) | 35.3 (10.3) | 69% | 60% | 32.2 (8.4) | 24.3 (5.3) | 25.9 (4.3) | NR | NR | 81% | 93% |
| Miller *et al*. (2014) | Fukuda (1994) | 18 | 41 | 44.2 (11.1) | 47.2 (9.2) | 88.9% | 80% | NR | 28.1 (4.6) | 26.7 (5.1) | NR | NR | 100% | 100% |
| Mueller *et al.* (2019) | Fukuda (1994) | 15 | 15 | 40.27 (8.84) | 40.80 (9.22) | 100% | 100% | NR | NR | NR | NR | NR | NR | NR |
| Murrough *et al.* (2010) | Fukuda (1994) | 17 | 19 | 47.9 (9.3) | 37.2 (13.8) | 88.2% | 52.6% | NR | 23.9 (2.7) | 26.2 (4.4) | NR | NR | 88.2% | 94.7% |
| Nakatomi *et al*. (2014) | Fukuda (1994) | 9 | 10 | 38.4 (5.1) | 39.1 (6) | 67% | 70% | 5.2 (7.3) | NR | NR | NR | NR | 100% | 100% |
| Neu *et al*. (2011) | Fukuda (1994) | 15 | 16 | 36.73 (7.1) | 36.94 (6.1) | 100% | 100% | NR | 23.33 (4.1) | 20.86 (1.9) | NR | NR | 100% | 100% |
| Neu *et al.* (2014) | Fukuda (1994) | 52 | 25 | 44.80 (1.9) | 40.46 (2.4) | 88.5% | 72% | NR | 24.5 (0.9) | 24.71 (1.1) | NR | NR | NR | NR |
| Okada *et al.* (2004) | Fukuda (1994) | 16 | 49 | 34 (24-46) | 34.4 (21-47) | 37.5% | 44.9% | 5.8 | NR | NR | NR | NR | NR | NR |
| Puri *et al.* (2002) | Fukuda (1994) | 8 | 8 | 42.7 (8.4) | 40.1 (8.8) | 75% | 50% | NR | NR | NR | NR | NR | NR | NR |
| Puri *et al.* (2012) | Fukuda (1994) | 26 | 26 | 42.9 (2.2) | 38.2 (2.2) | 73% | 73% | 10.9 (1.7) | NR | NR | NR | NR | NR | NR |
| Schmaling *et al*. (2003) | Fukuda (1994) | 15 | 15 | 44.4 (8.35) | 44.4 (8.35) | 80% | 80% | NR | NR | NR | NR | NR | NR | NR |
| Shan *et al.* (2016) | Fukuda (1994)  Canadian criteria (2011) | 15 | 10 | 34.06 (8.77) | 30.5 (7.93) | 73.3% | 80% | NR | NR | NR | NR | NR | 100% | 100% |
| Shan *et al.* (2017) | Canadian criteria (2011) | 38 | 14 | 34.8 (10.1) | 34.7 (8.4) | 71.0% | 71.4% | NR | NR | NR | NR | NR | NR | NR |
| Shan *et al.* (2018A) | Fukuda (1994) | 45 | 27 | 47.12 (11.67) | 43.10 (13.77) | 73.3% | 66.67% | NR | 26.58 (5.53) | 25.28 (4.18) | NR | NR | NR | NR |
| Shan *et al.* (2018B) | Fukuda (1994) | 43 | 26 | 47.4 (11.8) | 43.4 (13.9) | 72% | 69% | NR | 26.05 (5.04) | 24.83 (3.52) | NR | NR | NR | NR |
| Sherlin *et al*. (2007) | Fukuda (1994) | 17 | 17 | 40.6 | 40.6 | 88% | 88% | 7.4 | NR | NR | NR | NR | NR | NR |
| Shungu *et al*. (2012) | Fukuda (1994) | 15 | 13 | 32.7 (8.6) | 27.6 (7.4) | 80% | 53.8% | 9.7 (9.1) | 22.8 (3.2) | 23.0 (2.9) | NR | NR | NR | NR |
| Siessmeier *et al*. (2003) | Fukuda (1994) | 26 | 18 | 43 (9.3) | 38 (11.5) | 50% | 33% | NR | NR | NR | NR | NR | NR | NR |
| Staud *et al.* (2018) | Fukuda (1994)  Canadian criteria (2011) | 17 | 16 | 49.25 (11.43) | 49.60 (10) | 94.1% | 100% | NR | NR | NR | NR | NR | NR | NR |
| Tanaka *et al.* (2006) | Fukuda (1994) | 6 | 7 | 30.4 (4.8) | 26.1 (6.3) | 0% | 0% | 2.0 (1.3) | NR | NR | NR | NR | 100% | 100% |
| van der Schaaf *et al*. (2017) | Fukuda (1994) | 89 | 26 | 33.4 (1.2) | 32.8 (2.1) | 100% | 100% | 6.1 (0.7) | 25.1 (0.5) | 23.6 (0.8) | NR | NR | NR | NR |
| van der Schaaf *et al*. (2018) | Fukuda (1994) | 94 | 30 | 33.9 (SE: 1.2) | 33.4 (SE: 2) | 100% | 100% | 6.4 (SE: 0.8) | NR | NR | NR | NR | NR | NR |
| Vuong *et al.* (2019) | Fukuda (1994) | CFS (TMD+): 16  CFS (TMD-): 26 | 10 | CFS (TMD+):  43.9  CFS (TMD-):  46.7 | 49.4 | CFS (TMD+):  93.75%  CFS (TMD-): 65.4% | 70% | CFS (TMD+): 12.1 (10.4)  CFS (TMD-): 15.1 (10.2) | NR | NR | NR | NR | NR | NR |
| Wu *et al*. (2016) | Fukuda (1994) | 24 | 23 | 33.5 (1.92) | 33.3 (1.68) | 50% | 52.2% | NR | NR | NR | NR | NR | NR | NR |
| Yamamoto *et al*. (2012) | Fukuda (1994) | 10 | 10 | 35.7 (8.0) | 36.9 (10.1) | 60% | 50% | 6.9 (4.8) | NR | NR | NR | NR | NR | NR |
| Yamamoto *et al*. (2004) | Fukuda (1994) | CFS (+): 5  CFS (-): 6 | 11 | CFS (+): 39.2 (7.0)  CFS (-): 32.0 (2.5) | 32.9 (6.5) | CFS (+): 60%  CFS (-): 50% | 45.5% | NR | NR | NR | NR | NR | NR | NR |
| Zeinah *et al*. (2015) | Fukuda (1994) | 15 | 14 | 46.5 (13.2) | 46.6 (14.6) | 53.3% | 57.1% | 12.1 (6.9) | NR | NR | NR | NR | 86.7% | 71.4% |
| Zinn *et al.* (2016) | Canadian Criteria (2003) | 9 | 9 | 42.4 (20.5) | 42.4 (20.5) | 83.3% | 83.3% | NR | NR | NR | NR | NR | 94.4% right-handed in total no. of participants (n=18). | |
| Zinn *et al*. (2017) | Fukuda (1994) | 14 | 15 | 57.71 (15.15) | 31.13 (15.63) | 78.6% | 73.33% | NR | NR | NR | NR | NR | 100% | 93.3% |
| Zinn *et al*. (2018) | Fukuda (1994) | 50 | 50 | 51.8 (11.5) | 52.1 (11.5) | 76% | 76% | NR | NR | NR | NR | NR | NR | NR |

BMI, body mass index; Dx, diagnostic criteria; HC, healthy controls; ME/CFS, myalgic encephalomyelitis/ chronic fatigue syndrome; NR, not recorded; SD, standard deviation; TMD, temporomandibular disorders
