# Supplementary material for: A systematic review of neurological impairments in myalgic encephalomyelitis/ chronic fatigue syndrome using neuroimaging techniques
Source: PLoS One. 2020 Apr 30;15(4):e0232475. doi: 10.1371/journal.pone.0232475 (PMC7192498; doi:10.1371/journal.pone.0232475)
Supplement: S5 File — (DOCX) [file pone.0232475.s005.docx]

**S5.** Summary of primary outcome results table

| Author (year) | Imaging technique | Main study finding |
| --- | --- | --- |
| Armitage *et al*. (2009) | EEG/PSG | There was no significant difference in REM latency, delta-wave, fast frequency beta or alpha power in ME/CFS-afflicted twin cohort compared with the healthy twin cohort. ME/CFS cohort also had comparable PSG results with the healthy cohort. All of the following produced F ratios <1.0. These findings suggest that there is no evidence of sleep PSG and microarchitecture changes in ME/CFS. There, however, were significant differences in sleep stage main effect (p<0.001), EEG frequency band main effect (p < 0.001) and sleep stage by frequency band interaction (p< 0.0001) in ME/CFS patients compared to the healthy cohort. |
| Barnden *et al*. (2011) | T1w and T2w 1.5T MRI | An inverse correlation of increasing fatigue duration with decreased midbrain white matter volume was found in ME/CFS patients compared to HCs. In T1 weighted MRI, the group membership × hemodynamic score interactions were strongest in the midbrain GM of the brain stem, prefrontal WM, the caudal basal pons and hypothalamus. There was also a strong correlation found in ME/CFS between GM volume and pulse pressure (p=<0.001). |
| Barnden *et al*. (2015) | T1w and T2w 1.5T MRI | T1w signal in the ventrolateral thalamus (p<0.005), internal capsule (p<0.005) and prefrontal WM (p<0.001) is positively correlated with increasing ME/CFS severity. Upregulation of prefrontal myelin was reported in ME/CFS patients compared to HCs independent of anxiety and depression. The T2w signal when associated with ME/CFS illness duration demonstrated changes in the right middle temporal lobe, which is related to cognitive function (p<0.005). |
| Barnden *et al*. (2016) | T1w and T2w 1.5T MRI | When anxiety and depression were controlled for abnormal regressions were detected in the nuclei of the brainstem vasomotor centre, midbrain reticular formation, prefrontal white matter and the hypothalamus. The regulatory nuclei involved in stress response also showed abnormal regressions (p<0.05). There were no group differences detected on MRI. |
| Barnden *et al*. (2018) | T1w and T2w 1.5T MRI | Decreased signal levels in ME/CFS in a brainstem region were identified by T1w spin echo (p<0.002). There was also increased signal-levels in large bilateral clusters in sensorimotor cortex WM (p<0.0001). No differences in regional GM or WM volumes were found. |
| Biswal *et al*. (2011) | ASL 3T fMRI | ME/CFS patients showed a significant decrease in CBF in compared with HCs, including the left frontal (p = 0.0028), right frontal (p=0.0048), left parietal (p=0.0110), right parietal (p=0.0097), left temporal (p=0.0210), right temporal (p=0.0106). Nine of 11 patients studied showed a significant decrease in global CBF in ME/CFS patients compared with HCs, The remaining two ME/CFS patients showed an increase in CBF relative to HCs. |
| Boissoneault *et al*. (2016) | ASL 3T fMRI | ME/CFS patients had higher functional connectivity compared to HCs in selected regions, including: bilateral superior frontal gyrus, anterior cingulate cortex, precuneus, and right angular gyrus to precuneus, right postcentral gyrus, supplementary motor area, posterior cingulate gyrus, and thalamus.  ME/CFS patients had lower functional connectivity compared with HCs in the left parahippocampal gyrus, bilateral pallidum to right insula, right precentral gyrus and hippocampus. The reduced connectivity of the left parahippocampal gyrus positively correlated with clinical fatigue ratings (p<0.05). |
| Boissoneault *et al*. (2018) | ASL 3T fMRI | ME/CFS patients showed greater sFC inferior frontal gyrus connectivity to cerebellum, occipital, and temporal structures compared to HCs. In contrast, HCs showed greater increases in sFC between insula and temporo-occipital structures and between precuneus and thalamus/ striatum than ME/CFS patients. Patients also had lower dFC between hippocampus and right superior parietal lobule. dFC (p=0.004) and sFC (p=0.001) correlate with task related fatigue. |
| Boissoneault *et al*. (2019) | ASL 3T fMRI | There were no group differences detected in CBF or HRV. There was an inverse correlation between CBFV, HRV and fatigue symptoms (p = 0.001). Protective effect of high CBFV were highest in participants with low HRV. |
| Caseras *et al*. (2006) | T2w 1.5T fMRI | Overall performance in tasks were high in both groups and both activated the working memory network. Under 1-back condition, patients with ME/CFS displayed greater activation compared to HCs in the medial prefrontal regions, including the anterior cingulate gyrus (p<0.0033). When challenged, patients with ME/CFS had reduced activation in dorsolateral prefrontal and parietal cortices. In the 2- and 3-back conditions, ME/CFS had significantly higher activation of a large cluster in the right inferior/medial temporal cortex. As task load increased there were also significant differences in brain activation in ME/CFS patients compared to HCs this includes reduced activation of the dorsolateral, prefrontal and parietal cortices in ME/CFS patients compared to HCs. |
| Caseras *et al*. (2008) | T2w BOLD 1.5T fMRI | When fatigue was induced, activation of the occipito-parietal cortex (p=0.0009), posterior cingulate gyrus (p=0.0002) and para-hippocampal gyrus (NR) were increased while activation of dorsolateral and dorsomedial prefrontal cortex were decreased in ME/CFS patients compared with HCs during fatigue-inducing scenarios. These observations were reversed under anxiety-provoking conditions. P-values for the following observations were not reported. |
| Chaudhuri *et al.* (2002) | 1.5T ^1^H MRS | Compared to HCs, there was significant increase (p <0.001) in the spectra from choline-containing compounds with respect to unsuppressed water peaks. |
| Cleare *et al*. (2005) | PET and [11C]WAY-100635 | Significantly reduced serotonin (5-HT_1A_) receptor binding potential particularly in hippocampus bilaterally (23% total reduction) in ME/CFS patients compared with HCs (p<0.001). |
| Cook *et al*. (2007) | BOLD 3T fMRI | A significant correlation between mental fatigue and brain activity in ME/CFS patients when instructed to complete a challenging working memory task and HCs were found. Brain regions of significance include: cerebellar, temporal lobe, cingulate gyrus, frontal lobe regions (positive relationship) (p ≤0.005) and left posterior parietal cortex (p=0.001). There was no statistical significance found between mental fatigue and brain activity when provided with a simple auditory monitoring task. |
| de Lange *et al*. (2004) | Rapid event- related 1.5T fMRI | While ME/CFS patients and HCs show use of overlapping neural resources including activation of dorsal anterior cingulate cortex during error trials, the ME/CFS cohort performance speed on motor tasks was considerably slower. ME/CFS showed greater activation of visually related structures (p < 0.05). The ventral anterior cingulate cortex was active in error trials in HCs, however, remained inactive in ME/CFS (p < 0.05). |
| de Lange *et al*. (2005) | VBM 1.5T MRI | Global GM was significantly reduced in both ME/CFS cohorts compared with HCs (p < 0.001). This decline in GM volume was correlated with a reduction in physical activity (P < 0.001). |
| Decker *et al*. (2009) | EEG/ PSG | Compared with HCs, alpha power in the ME/CFS cohort was diminished during stage 2, slow wave and REM sleep (p < 0.0001). Delta power decreased during slow wave sleep (p < 0.0001) but was elevated during stage 1 (p < 0.0001) and REM (p < 0.0001). Theta, sigma and beta spectral power during stage 2, slow wave sleep and REM were all significantly reduced in patients compared to their matched HCs (<0.0001). |
| Finkelmeyer *et al*. (2018A) | VBM 3T MRI | There is a negatively correlated relationship between intracranial compliance and cerebral perfusion in both ME/CFS patients and HCs and no significant differences were found in intracranial compliance in ME/CFS patients compared with HCs. Orthostatic intolerance (OI) symptoms were positively correlated with low intracranial compliance (p=0.033) and higher resting perfusion in ME/CFS patients (p=0.038). |
| Finkelmeyer *et al*. (2018B) | phase-contrast, 3T qfMRI | When total intracranial volume is accounted for, ME/CFS patients were found to have larger GM volume particularly in regions including the amygdala and insula and lower WM volume in regions such as the midbrain, pons and right temporal lobe compared with HCs (p<0.05). |
| Flor-Henry *et al.* (2010) | EEG | EEG source analysis to classify and separate HCs and ME/CFS was effective with a correct retrospective classification rate of 72% during rest to a maximum of 83% in the alpha band during the verbal cognitive condition. Significant differences between groups were found in both word finding and dot localisation cognitive conditions. This includes elevated source-current activity in the left frontal-temporal-parietal regions compared with HCs (p<0.05). |
| Gay *et al.* (2016) | 3T MRI | Disrupted interregional RS FC was identified in ME/CFS patients using data-driven and model-based analyses. Five resting state networks were analysed: DMN, SN, LFPN, right frontoparietal networks and SMN. Decreased RS FC was identified across all networks for ME/CFS patients compared with HC (p<0.001). There is an observable positive correlation between altered RS FC and self- reported fatigue (p<0.05). |
| Kim *et al*. (2015) | 3T fMRI | Posterior cingulate cortex showed increased RS FC with the dorsal and rostral anterior cingulate cortex in the ME/CFS cohort compared with HCs (p<0.001). Connectivity strength of posterior cingulate nucleus with dorsal cingulate nucleus was correlated with Chalder Fatigue scale score (p<0.050). Overall, the global efficiency of the posterior cingulate cortex was significantly lower in ME/CFS patients compared with HC and there was no difference in local efficiency of brain regions for ME/CFS patients when compared with HCs. |
| Lange *et al.* (2005) | BOLD 1.5T fMRI | ME/CFS had comparable auditory information processing performance as HCs; however, to achieve this, more of the network associated with the working system becomes activated. ME/ CFS patients experience significantly greater mental fatigue compared to HCs (p<0.001) |
| Le Bon *et al.* (2012) | EEG | The ultra-slow delta power was approximately one fifth lower in ME/CFS patients compared to HCs in N3 sleep (p=0.046). The other frequency bands measured: theta, alpha, sigma and beta did not differ significantly between the two groups. |
| Lewis *et al*. (2001) | fMRI SPECT | No significant differences in resting regional CBF (rCBF) values were found between the healthy twin cohort and the ME/CFS-afflicted twin cohort. |
| Mathew *et al.* (2008) | T1w MRI 3T ^1^H MRS | Concentrations of ventricular lactate in ME/CFS patients were significantly increased (p <0.001) compared to HCs. |
| Miller *et al*. (2014) | T2w 3T fMRI | A significant decrease in activation of the right caudate nucleus (p = 0.01) and right globus pallidus (p = 0.02) was found in ME/CFS patients compared with HCs. When measured by the multidimensional fatigue inventory, the decreased activation in the globus pallidus was significantly correlated with increased mental fatigue (p=0.001), general fatigue (p=0.01) and reduced activity (p=0.02). |
| Mueller *et al.* (2019) | 3T MRS - whole-brain echo-planar spectroscopic imaging | There was a positive correlation between fatigue and metabolite ratios in seven regions in ME/CFS patients compared to HCs. In the left anterior cingulate, mean levels of choline respective to creatine was significantly higher in ME/CFS patients (p < 0.001). There was a temperature increase found in the right insula, putamen, frontal cortex, thalamus, and the cerebellum (p < 0.05). The temperature changes in the right insula, right thalamus and cerebellum were related to elevated LAC/CR ratios (p< 0.05). |
| Murrough *et al.* (2010) | ^1^H MRS | Concentrations of ventricular CSF lactate in ME/CFS patients were significantly increased compared to HCs (p=0.01). The increase in ventricular CSF lactate positively correlated with the severity of mental fatigue (r=0.63, p = 0.02). |
| Nakatomi *et al*. (2014) | C-(R)-PK11195 PET | When assessing the binding potential of 1C-(R)-PK11195, a ligand for a translocator protein in activated microglia or astrocytes that can be used as a marker for neuroinflammation binding, was significantly higher ranging from 45-199% in the cingulate cortex (p<0.04), us (p<0.02), amygdala (p<0.06), thalamus (p< 0.0013), midbrain (p <0.0001) and pons (p <0.0021) in ME/CFS patients compared to HCs. The highest binding potential was in the cingulate cortex with a 199% increase in ME/CFS patients. In the cingulate cortex and thalamus the binding potential value positively correlated with pain score. The binding potential in the hippocampus was positively related to the depression score. |
| Neu *et al*. (2011) | EEG P300 | The following tests were undertaken in ME/CFS patients and HCs to act as a cognitive and behavioural measure: the auditory verbal learning test (AVLT), digit span, digit symbol and finger tapping test (FTT) (p≤0.01). There was significantly lower performance in all tests for the ME/CFS cohort compared with HCs excluding the digit span and AVLT tests. |
| Neu *et al.* (2014) | EEG spectral analysis | ME/CFS patients presented with impaired subjective sleep quality and higher sleepiness levels compared with HCs. ME/CFS patients showed lower occipital ultra-slow delta power (US) and higher occipital theta and alpha power. The ME/CFS cohort also had lower central US and occipital US, and higher central delta power (p<0.001) |
| Okada *et al.* (2004) | VBM 3T MRI | ME/CFS patients had reduced GM in the bilateral prefrontal cortex compared with HCs as shown by reduced acetyl-L-carnitine uptake. Reduced GM in the right prefrontal cortex negatively correlated with symptom severity (p=0.004). |
| Puri *et al.* (2002) | 1.5T ^1^H MRS | The mean levels of choline respective to creatine was significantly higher in ME/CFS patients (p=0.008) compared to HCs. No other significant differences were found in the other metabolites tested: N-acetylaspartate and creatine. |
| Puri *et al.* (2012) | VBM 3T MRI study | Significantly lower GM volumes in ME/CFS patients compared with HCs were found in the occipital lobes (including the right and left occipital poles; left lateral occipital cortex; superior division; and left supracalcrine cortex), right angular gyrus and the posterior division of the left parahippocampal gyrus (p<0.05). Reduced WM volumes was detected in left occipital lobe in ME/CFS patients compared with HCs (p<0.05). |
| Schmaling *et al*. (2003) | fMRI SPECT | While performing a PASAT task, the anterior cingulate region showed less perfusion in ME/CFS patients compared with HCs. Activation of the left anterior cingulate region during the task, however, was much greater for the patients. There was no difference found in performance on the PASAT despite ME/CFS patients reporting exertion of more mental effort to perform a task compared with HCs (p<0.05). |
| Shan *et al.* (2016) | Longitudinal 1.5T MRI | Over time, there was a reported significant decrease in WM volume in the left inferior fronto-occipital fasciculus (IFOF) in ME/CFS patients compared with HCs. Group comparisons also showed that there were significantly decreased regional WM volumes in adjacent regions and decreased GM and blood volumes in contralateral regions of the IFOF for the ME/CFS cohort compared with HCs. These features positively correlated with ME/CFS symptom severity scores (p<0.05). |
| Shan *et al.* (2017) | T1w and T2w 1.5T MRI | ME/CFS had significantly lower magnetization transfer (MT) and T1W intensities compared with HCs (p<0.05). There was a negative correlation in the medial prefrontal cortex between pittsburgh sleep quality index and MT-T1W intensities in the ME/CFS cohort. |
| Shan *et al.* (2018A) | BOLD 3T fMRI | BOLD signals demonstrated increased complexity in the posterior cingulate cortex in the default mode network in both resting state and during a stroop task (p < 0.05). FC between medial prefrontal cortex and inferior parietal lobules were significantly weaker in the resting state (p <0.05) however was more complex in the ME/CFS group during the task (p < 0.05). |
| Shan *et al.* (2018B) | tfMRI | The activated regions were detected and measured using sample entropy (SampEN). ME/CFS patients had a longer response time to the task but no significant difference in accuracy compared with HC. BOLD SampENS was significantly lower in ME/CFS patients (p<0.05). The BOLD signal in the medioventral occipital cortex explained 40% and 31% of the variance in the SF-36 PCS and MCS scores respectively while the bold signal in the precentral gyrus explained 16% and 7% of the PCS and MCS scores respectively. |
| Sherlin *et al*. (2007) | EEG LORETA | Significant differences in current source density was detected in ME/CFS patients compared with the healthy twin cohort. ME/CFS patients were found to have higher delta power in the left uncus and parahippocampal gyrus (t score: 3.61). The cingulate gyrus and right superior frontal gyrus also had higher theta power in ME/CFS patients compared with the healthy twin cohort (t score: 6.08). |
| Shungu *et al*. (2012) | ASL fMRI  3T MRS | ME/CFS patients had higher venticular lactate and decreased glutathione (GSH) levels compared with HCs (p<0.001). The levels of ventricular lactate and cortical GSH were inversely correlated to physical health and disability scores. Patients also had lower rCBF in the left cingulate cortex (p = 0.039) and right lingual gyrus (p=0.016). No differences were found between the ME/CFS cohort and HC group in high- energy phosphate metabolites. |
| Siessmeier *et al*. (2003) | FDG-PET | Two ME/CFS patients showed hypometabolism in the cuneus/praecuneus and 12 patients showed hypometabolism bilaterally in the cingulate gyrus and adjacent mesial cortical areas compared with HCs (p <0.05). Five of these 12 patients also had decreased metabolism in the orbitofrontal cortex (p <0.001). The remaining 12 ME/CFS patients showed no significant decrease in FDG uptake compared with HCs. Correlation analysis revealed no link between regional reductions in glucose metabolism and fatigue, however, reduced glucose metabolism was inversely correlated with anxiety, depression (p <0.001) and health related quality of life (p<0.0001) measures. |
| Staud *et al.* (2018) | ASL 3T fMRI | At baseline, ME/CFS patients had higher reported fatigue levels compared with HCs (p<0.01). Both groups reported higher fatigue levels and increased CBF following the PASAT task, which then decreased after three minutes (p < 0.0001). During the post-task recovery period, there was a significant difference in rCBF values between patient groups and HCs (p<0.05). Improvement of fatigue symptoms in HCs was linked to increased rCBF in both superior temporal gyri, precuneus and fusiform gyri. In these areas in ME/CFS patients, rCBF values were decreased (p<0.0001). |
| Tanaka *et al.* (2006) | 3T fMRI | There was an equivalent decrease in responsiveness of task-dependent brain regions when provided with a fatigue-inducing task in both ME/CFS patients and HCs. The responses in the auditory cortices remained constant in the HCs, however, it was attenuated in ME/CFS patients; this was correlated with the subjective sensation of fatigue (p<0.01). |
| van der Schaaf *et al*. (2017) | 3T MRI | Global GM volume did not differ between ME/CFS patients and HCs. Pain symptoms were the most effective predictor of both GM volume and *N-acetyl*aspartylglutamate/ creatine (NAA/Cr) ratio in the left dorsolateral prefrontal cortex. |
| van der Schaaf *et al*. (2018) | MRI | Reduced feedback- related activity in the dorsolateral prefrontal cortex proportional to state -related fatigue and prior beliefs about task performance ability was found in ME/CFS patients compared to HCs (r=0.24, p=0.024). Additionally, ME/CFS patients demonstrated higher activity in the supplementary motor area (r=0.238, p=0.012) and reduced connectivity between this area and the sensorimotor cortex during motor preparation (p =0.006). |
| Vuong *et al.* (2019) | T1w 3T MRI | When provided with autonomic challenge HCs and both ME/CFS patients who did or didn’t screen positive for a temporomandibular disorder (TMD) showed increased brain activation in the superior and inferior frontal gyri, the left and right putamen, thalamus, and the insular cortex. ME/CFS patients who had a positive screening for a TMD showed increased activity in the left caudate nucleus compared to HCs (p < 0.05). |
| Wu *et al*. (2016) | EEG | In ME/CFS patients delta, theta, and alpha1 waves were significantly increased predominantly in the right frontal and left occipital regions compared with HCs (p<0.05). This indicates that there is region specific encephalic distribution. The correlation dimension in patients was also found to be lower compared with HCs. This feature is suggestive of reduced EEG complexity in ME/CFS patients. |
| Yamamoto *et al*. (2012) | 0.3T MRI  PET | ME/CFS patients that tested positive for serum autoantibodies against Muscarinic Cholinergic Receptor (mAChR) were found to have a reduction of (N-[11C]methyl-3-piperidyl benzilate [11C](+)3-MPB binding in brain regions, predominantly the dorsolateral prefrontal cortex (20%) (p<0.05), anterior cingulate cortex (21%) (p<0.05), orbitofrontal cortex (21%) (p<0.01) compared with HCs and ME/CFS patients that were negative for these autoantibodies. This feature did not alter acetylcholinesterase activity. |
| Yamamoto *et al*. (2004) | PET | A reduction of [“C] (+) MCN5652 radiotracer used to detect levels of serotonin transporter (5-HTTs) was identified in the anterior cingulate in ME/CFS patients compared to HCs indicating reduced density of 5-HTTs (p<0.05). |
| Zeinah *et al*. (2015) | T1w, DTI, ASL 3T MRI | FA was significantly increased in right articulate fasciculus in ME/CFS patients compared with HCs. An increase in FA in the inferior longitudinal fasciculus as also detected in dextrous participants. In ME/CFS patients, the increase in right anterior arculate FA positively correlated with disease severity. Bilateral WM atrophy was also detected in ME/CFS patients compared with HCs. An increase in cortical thickness in both orculate end points, middle temporal and precentral gyri were also detected in ME/CFS patients. ASL showed no significant differences between groups. |
| Zinn *et al.* (2016) | eLORETA EEG | There was evidence of hypoconnectivity in the delta, alpha and alpha-2 frequency bands in ME/CFS patients compared with HCs. Additionally, there was detection of resting state dysfunction in the occipital, parietal, posterior temporal and posterior cingulate in the ME/CFS cohort. Disrupted connectivity of the CEN (p=0.024), SN (p=0.037) and DMN (0.021) were positively correlated with cognitive impairment in ME/CFS patients. |
| Zinn *et al*. (2017) | qEEG | In ME/CFS patients, small-worldness for the delta band was significantly lower compared with HCs. This delta small worldness results in a greater risk of complex brain network inefficiency (p < 0.05). There was a negative correlation between small worldness and neurocognitive impairment scores in ME/CFS patients on the De paul symptom questionaire (DSQ). |
| Zinn *et al*. (2018) | eLORETA qEEG | The delta (1-3Hz) (p=0.028) and beta-2 (19-21Hz) (p=0.024) frequency bands were significantly different in ME/CFS patients and HCs. Delta was increased in patient groups while beta-2 current density was lower. These delta sources were predominantly identified in the frontal lobe. Beta-2 sources were identified in the medial and superior lobe. There was a negative correlation identified between a clinical reduction in motivation and left-laterized frontal delta sources (p=0.008). |

ASL, arterial spin labelling; AVLT, auditory verbal learning test; BOLD, blood oxygenation level dependent; CBF, cerebral blood flow; ME/CFS, myalgic encephalomyelitis/ chronic fatigue syndrome; DMN, default mode network; DX, diagnostic criteria; EEG, electroencephalogram; eLORETA, exact low-resolution brain electromagnetic tomography; dFC, dynamic functional connectivity; FTT, finger tapping task; FC, functional connectivity; fMRI, functional magnetic resonance imaging; GM, gray matter; HRV, heart rate variability; HCs, healthy controls; ^1^H MRS, proton magnetic resonance spectroscopy; LFPN, left frontoparietal networks; MRI, magnetic resonance imaging; PCS, physical component summary; MRS, magnetic resonance spectroscopy; MT, magnetic transfer; MCS, mental component summary; mAChR, muscarinic cholinergic receptor; NR, not recorded; PASAT, Paced Auditory Serial Addition Test; PSG, polysomnography; PET, positron emission tomography; qfMRI; quantitative functional magnetic resonance imaging; REM, rapid eye movement; RS, resting state; SN, salience network; SMN, sensory motor network; SPECT, single-photon emission computed tomography; sFC, static functional connectivity; T1W, T1-weighted; T2W- weighted; VBM, voxel-based morphometry; WM, white matter; 5-HTTs, serotonin transporters
