# Supplementary material for: A systematic review of neurological impairments in myalgic encephalomyelitis/ chronic fatigue syndrome using neuroimaging techniques
Source: PLoS One. 2020 Apr 30;15(4):e0232475. doi: 10.1371/journal.pone.0232475 (PMC7192498; doi:10.1371/journal.pone.0232475)
Supplement: S6 File — (DOCX) [file pone.0232475.s006.docx]

**S6.** Secondary outcome measures table

| Author | Secondary outcome measure (s) | Results |
| --- | --- | --- |
| Armitage *et al*, 2009 | NA | NA |
| Barnden *et al*. 2011 | Bell score  Total SS  HADS depression  HADS anxiety | Bell score: <0.0001^a^  Total SS: <0.0001^a^  HADS depression: <0.0002^a^  HADS anxiety: <0.0001^a^ |
| Barnden *et al*. 2015 | HADS depression  HADS anxiety | NR |
| Barnden *et al*. 2016 | HADS depression  HADS anxiety | HADS depression: <0.0002 ^a^  HADS anxiety: <0.0001^a^ |
| Barnden *et al*. 2018 | NA | NA |
| Boissoneault *et al*. 2016 | VAS anxiety  VAS fatigue  VAS Pain  VAS Depression  VAS PILL total score  VAS Physical function  VAS Role function | VAS anxiety: p<0.05 ^a^  VAS fatigue: p<0.05 ^a^  VAS Pain: p<0.05^a^  VAS Depression: p<0.05^a^  VAS PILL total score: p<0.05^a^  VAS Physical function: p<0.05^b^  VAS Role function: p<0.05^b^ |
| Boissoneault *et al*. 2018 | VAS anxiety  VAS fatigue  VAS Pain  VAS Depression  VAS PILL total score  VAS Physical function  VAS Role function | VAS anxiety: p<0.05 ^a^  VAS fatigue: p<0.05 ^a^  VAS Pain: p<0.05^a^  VAS Depression: p<0.05^a^  VAS PILL total score: p<0.05^a^  VAS Physical function: p<0.05^b^  VAS Role function: p<0.05^b^ |
| Boissoneault *et al*. 2019 | VAS anxiety  VAS fatigue  VAS Pain  VAS Depression  VAS PILL total score  VAS Physical function  VAS Role function | VAS anxiety: p<0.05 ^a^  VAS fatigue: p<0.05 ^a^  VAS Pain: p<0.05^a^  VAS Depression: p<0.05^a^  VAS PILL total score: p<0.05^a^  VAS Physical function: p<0.05^b^  VAS Role function: p<0.05^b^ |
| Caseras *et al*. 2006 | ChFS  WSAS  HADS depression  HADS anxiety  PF-SF36 | ChFS: p<0.001 ^a^  WSAS: 0 ^a^  HADS depression: p<0.001 ^a^  HADS anxiety: p<0.001 ^a^  PF-SF36: p<0.001^b^ |
| Caseras *et al*. 2008 | ChFS  WSAS  HADS depression  HADS anxiety  PF-SF36 | ChFS: p<0.001 ^a^  WSAS: 0 ^a^  HADS depression: p<0.001 ^a^  HADS anxiety: p<0.001 ^a^  PF-SF36: p<0.001^b^ |
| Chaudhuri *et al*. 2003 | NA | NA |
| Cleare *et al*. 2005 | GHQ-12 score  BDI score  Spielberger Trait Anxiety Score  Spielberger State Anxiety Score  Disability Score | Not reported for HCs |
| Cook *et al*. 2007 | BDI score  State Anxiety Score (STAI) | BDI score: d =1.8 ^a^  State Anxiety Score (STAI): d= 1.1 ^a^ |
| de Lange *et al*. 2004 | **CIS-R**  Fatigue  Reduced concentration  Reduced motivation  Reduced activity  SIP-8 total  SCK089  BDI  BDI- primary care | Statistical analysis not conducted. |
| de Lange *et al*. 2005 | **CIS-R**  Fatigue  Reduced concentration  Reduced motivation  Reduced activity | NR |
| Decker *et al*. 2009 | NA | NA |
| Finkelmeyer *et al*. 2018A | SF-36 physical health  SF-36 mental health | SF-36 physical health: p <0.001 ^a^  SF-36 mental health: p <0.001 ^a^ |
| Finkelmeyer *et al*. 2018B | NA | NA |
| Flor-Henry *et al*. 2010 | NA | NA |
| Gay *et al*. 2016 | MFI General fatigue  MFI Physical Fatigue  MFI Reduced Activity  MFI Reduced motivation  MFI fatigue | MFI General fatigue: p <0.01^a^  MFI Physical Fatigue: p <0.01^a^  MFI Reduced Activity: p <0.01^a^  MFI Reduced motivation: p <0.01^a^  MFI fatigue: : p <0.01^a^ |
| Kim *et al*. 2015 | Chalder fatigue  PGWBI score  BDI score  BAI score | Chalder fatigue: p <0.0001^a^  PGWBI score: p <0.0001^a^  BDI score: p <0.0001^a^  BAI score: p <0.0001^a^ |
| Lange *et al*. 2005 | NA | NA |
| Le Bon *et al* 2012 | PSQI  FSS  ESS | PSQI: p <0.001^a^  FSS: p <0.001^a^  ESS: p <0.010^a^ |
| Lewis *et al*. 2001 | NA | NA |
| Mathew *et al*. 2009 | FSS  HAM-A  PSWQ  HAMD  PSQI | FSS: p<0.001  HAM-A: p<0.05  PSWQ: p<0.001  HAMD: p=0.42  PSQI: NR |
| Miller *et al*. 2014 | SF-36 bodily pain  SF-36 General health  SF-36 Mental health  SF-36 physical functioning  SF-36 role emotional  SF-36 role physical  SF-36 social functioning  SF-36 vitality | SF-36 bodily pain: p <0.0001^a^  SF-36 General health: p <0.0001^a^  SF-36 Mental health: p <0.0001^a^  SF-36 physical functioning: p <0.0001^a^  SF-36 role emotional: p <0.0005^a^  SF-36 role physical: p <0.005^a^  SF-36 social functioning: p <0.0001^a^  SF-36 vitality: p <0.0001^a^ |
| Mueller *et al*. 2019 | NA | NA |
| Murrough *et al*. 2010 | General fatigue  Physical fatigue  Mental fatigue  Reduced activity  Reduced motivation  HAMD | General fatigue: p <0.0001^a^  Physical fatigue: p <0.0001^a^  Mental fatigue: p <0.0001^a^  Reduced activity: p <0.0001^a^  Reduced motivation: p <0.0001^a^  HAMD: p <0.0001^a^ |
| Nakatomi *et al*. 2014 | VAS of fatigue sensation  Chalder fatigue scale  Cognitive impairment  Pain  CES-D | VAS of fatigue sensation: p <0.0074^a^  Chalder fatigue scale: p <0.0011^a^  Cognitive impairment: p <0.0013^a^  Pain: p <0.0001^a^  CES-D: p <0.0020^a^ |
| Neu *et al*. 2011 | HAMD  HAMA  BDI-13  PSQI  FSS  ESS | HAMD: p <0.0001^a^  HAMA: p <0.0001^a^  BDI-13: p <0.0001^a^  PSQI: p <0.0001^a^  FSS: p <0.0001^a^  ESS: <0.0001^a^ |
| Neu *et al*. 2014 | PSQI  ESS  FSS  HAD-A  HAD-D | PSQI: p <0.001^a^  ESS: p <0.001^a^  FSS: p <0.001^a^  HAD-A: p <0.001^a^  HAD-D: p <0.001^a^ |
| Okada *et al*. 2004 | NA | NA |
| Puri *et al*. 2002 | NA | NA |
| Puri *et al*. 2012 | NA | NA |
| Schmaling *et al*. 2003 | SF-36 physical functioning  MASQ | SF-36 physical functioning: p < 0.001^a^  MASQ: p < 0.001^a^ |
| Shan *et al*. 2016 | Bell score  Depression  Anxiety  Neuro SS  Somatic SS | Bell score: p <0.0062^a^  Depression: p <0.0062^a^  Anxiety: p <0.0062^a^  Neuro SS: p <0.0062^a^  Somatic SS: p <0.0062^a^ |
| Shan *et al*. 2017 | CFQ  PSQI  Anxiety  Depression | CFQ: p <0.008^a^  PSQI: p <0.008^a^  Anxiety: p <0.008^a^  Depression: p <0.008^a^ |
| Shan *et al*. 2018A | SF-36 PCS  SF-36 MCS | SF-36 PCS: p <0.001^a^  SF-36 MCS: p <0.001^a^ |
| Shan *et al*. 2018B | SF-36 PCS  SF-36 MCS | SF-36 PCS: p <0.001^a^  SF-36 MCS: p <0.001^a^ |
| Sherlin *et al*. 2007 | Current depression | Current depression: p=0.046^a^ |
| Shungu *et al*. 2012 | CDC  SDS  QIDS  WBPI  PSQI  Total CTQ  RAND total  MFI RA  MFI MF  MFI PF  MFI RM  MFI GF | CDC: p <0.001^a^  SDS: p <0.001^a^  QIDS: p <0.001^a^  WBPI: p <0.001^a^  PSQI: p <0.001^a^  Total CTQ: p <0.001^a^  RAND total: p <0.001^a^  MFI RA: p <0.001^a^  MFI MF: p <0.003^a^  MFI PF: p <0.001^a^  MFI RM: p <0.058^a^  MFI GF: p <0.001^a^ |
| Sevel *et al*. 2018 | **VAS rating**  Fatigue  Sleep quality  Pain intensity  Depression  Anxiety  Anger | **VAS rating**  Fatigue: p < .0001 ^a^  Sleep quality: p < .0001 ^a^  Pain intensity: p < .0001 ^a^  Depression: p= 0.001 ^a^  Anxiety: p < .0001 ^a^  Anger: p= 0.006 ^a^ |
| Siessmeier *et al*. 2003 | Anxiety  Depression  HRQOL physical  HRQOL mental  Fatigue | Statistical tests not conducted. |
| Staud *et al*. 2018 | VAS fatigue  VAS pain  VAS anxiety  VAS Depression | VAS fatigue: p <0.001^a^  VAS pain: p <0.001^a^  VAS anxiety: p <0.001^a^  VAS Depression: p <0.001^a^ |
| Tanaka *et al*. 2006 | VAS fatigue | VAS fatigue: p <0.001^a^ |
| van der Schaaf *et al*. 2017 | CIS- fatigue  SF-36  BDI-PC | CIS- fatigue: p <0.001^a^  SF-36a: p <0.001^a^  BDI-PC: p <0.001^a^ |
| van der Schaaf *et al*. 2018 | CIS- fatigue  SIP-total  SF-36  BDI-PC | CIS- fatigue: p <0.001^a^  SIP-total: NA  SF-36a: p <0.001^a^  BDI-PC: p <0.001^a^ |
| Vuong *et al.* 2019 | DSQ  FIS  PCQ | DSQ ^b^  FIS ^b^  PCQ ^b^ |
| Wu *et al*. 2016 | HAMD  HAMA | Statistical tests not conducted. |
| Yamamoto *et al*. 2004 | VAS fatigue | VAS fatigue: p <0.001^a^ |
| Yamamoto *et al*. 2012 | VAS fatigue  Attention score  Pain score | VAS fatigue: p <0.0001^a^  Attention score: p <0.0005^a^  Pain score: p <0.0005^a^ |
| Zeineh *et al*. 2015 | MFI-20 | MFI-20: p <0.001^a^ |
| Zinn *et al*. 2016 | NA | NA |
| Zinn *et al*. 2017 | DSQ cognitive composite score | DSQ cognitive composite score: p <0.01^a^ |
| Zinn *et al*. 2018 | MFI-20 total score  MFI-20 general fatigue  MFI-20 physical fatigue  MFI-20 reduced activity  MFI-20 reduced motivation  MFI-20 mental fatigue  FSS score | MFI-20 total score: p <0.001^a^  MFI-20 general fatigue: p <0.001^a^  MFI-20 physical fatigue: p <0.001^a^  MFI-20 reduced activity: p <0.001^a^  MFI-20 reduced motivation: p <0.001^a^  MFI-20 mental fatigue: p <0.001^a^  FSS score: p <0.001^a^ |

BDI, Beck Depression Inventory; CDC, center of disease control; CTQ, childhood trauma questionaire; CIS-R, Clinical interview schedule – revised; DSQ, disability-screening questionaire; ESS, European social survey; HAMD, hamilton rating scale depression; HAMA, hamilton rating scale anxiety; HRQOL, health related quality of life; FSS, fatigue severity scale; HADS, hospital anxiety and depression scale; MFI, multidimensional fatigue inventory; NA, not applicable; NR, not recorded; RAND, VAS, visual analogue scale; SF-36, 36-item short form survey; SS, symptom score. ^a^= ME/CFS > HC, ^b^= Comparison with HC not made.
