# Supplementary material for: A systematic review of neurological impairments in myalgic encephalomyelitis/ chronic fatigue syndrome using neuroimaging techniques
Source: PLoS One. 2020 Apr 30;15(4):e0232475. doi: 10.1371/journal.pone.0232475 (PMC7192498; doi:10.1371/journal.pone.0232475)
Supplement: S7 File — (DOCX) [file pone.0232475.s007.docx]

**S7.** JBI quality assessment table and descriptions

**Quality assessment**

|  | 1 | 2 | 3 | 4 | 5 | 6 | 7 | 8 | 9 | 10 |
| --- | --- | --- | --- | --- | --- | --- | --- | --- | --- | --- |
| Armitage et al.2009 | Yes | Yes | Yes | N/A | N/A | Yes | Yes | Yes | N/A | No |
| Barnden et al. 2011 | Yes | No | Yes | N/A | N/A | Yes | Yes | Yes | N/A | Yes |
| Barnden et al. 2015 | Yes | No | Yes | N/A | N/A | Yes | Yes | Yes | N/A | Yes |
| Barnden et al. 2016 | Yes | No | Yes | N/A | N/A | Yes | Yes | Yes | N/A | Yes |
| Barnden et al. 2018 | No | No | Yes | N/A | N/A | Yes | Yes | Yes | N/A | No |
| Biswal et al. 2011 | No | No | No | N/A | N/A | No | No | Yes | N/A | No |
| Boissoneault et al. 2016 | Yes | No | Yes | N/A | N/A | Yes | Yes | Yes | N/A | No |
| Boissoneault et al. 2018 | Yes | No | Yes | Yes | Yes | Yes | Yes | Yes | Yes | No |
| Boissoneault et al. 2019 | Yes | No | Yes | N/A | N/A | Yes | Yes | Yes | N/A | No |
| Caseras et al. 2006 | Yes | Yes | Yes | Yes | Yes | Yes | Yes | Yes | Yes | Yes |
| Caseras et al. 2008 | Yes | Yes | Yes | Yes | Yes | Yes | Yes | Yes | Yes | Yes |
| Chaudhuri et al. 2003 | Yes | No | No | N/A | N/A | Yes | Yes | Yes | N/A | Yes |
| Cleare et al. 2005 | Yes | No | U | Yes | No | Yes | Yes | Yes | Yes | Yes |
| Cook et al. 2007 | Yes | Yes | Yes | Yes | Yes | Yes | Yes | Yes | Yes | Yes |
| de Lange et al. 2004 | Yes | No | Yes | Yes | Yes | Yes | Yes | Yes | Yes | Yes |
| de Lange et al. 2005 | Yes | No | U | N/A | N/A | Yes | Yes | Yes | N/A | Yes |
| Decker et al. 2009 | Yes | Yes | Yes | N/A | N/A | Yes | Yes | Yes | N/A | No |
| Finkelmeyer et al. 2018A | Yes | Yes | Yes | N/A | N/A | Yes | Yes | Yes | N/A | No |
| Finkelmeyer et al. 2018B | Yes | Yes | Yes | N/A | N/A | Yes | Yes | Yes | N/A | Yes |
| Flor-Henry et al. 2010 | Yes | No | Yes | Yes | Yes | Yes | Yes | Yes | Yes | Yes |
| Gay et al. 2016 | Yes | Yes | Yes | N/A | N/A | No | No | Yes | N/A | Yes |
| Kim et al. 2015 | Yes | Yes | Yes | N/A | N/A | Yes | Yes | Yes | N/A | No |
| Lange et al. 2005 | Yes | No | Yes | Yes | Yes | Yes | Yes | Yes | Yes | Yes |
| Le Bon et al 2012 | Yes | U | Yes | N/A | N/A | Yes | Yes | Yes | N/A | No |
| Lewis et al. 2001 | Yes | Yes | Yes | Yes | Yes | Yes | Yes | Yes | Yes | No |
| Mathew et al. 2008 | Yes | No | Yes | N/A | N/A | Yes | Yes | Yes | N/A | Yes |
| Miller et al. 2014 | Yes | Yes | Yes | Yes | Yes | Yes | Yes | Yes | Yes | No |
| Mueller et al. 2019 | Yes | No | Yes | N/A | N/A | Yes | Yes | Yes | N/A | Yes |
| Murrough et al 2010 | Yes | No | Yes | N/A | N/A | Yes | Yes | Yes | N/A | Yes |
| Nakatomi et al. 2014 | Yes | No | Yes | Yes | Yes | Yes | Yes | Yes | Yes | Yes |
| Neu et al. 2011 | Yes | No | Yes | N/A | N/A | Yes | Yes | Yes | N/A | No |
| Neu et al. 2014 | Yes | No | No | N/A | N/A | Yes | Yes | Yes | N/A | Yes |
| Okada et al. 2004 | U | Yes | No | N/A | N/A | Yes | Yes | Yes | N/A | Yes |
| Puri et al. 2002 | Yes | No | Yes | N/A | N/A | No | No | Yes | N/A | No |
| Puri et al. 2012 | Yes | No | Yes | N/A | N/A | No | No | Yes | N/A | Yes |
| Schmaling et al. 2003 | Yes | Yes | Yes | Yes | Yes | Yes | Yes | Yes | Yes | No |
| Shan et al. 2016 | No | No | Yes | N/A | N/A | Yes | Yes | Yes | N/A | Yes |
| Shan et al. 2017 | Yes | No | Yes | N/A | N/A | Yes | Yes | Yes | N/A | Yes |
| Shan et al. 2018 | Yes | No | Yes | Yes | Yes | Yes | Yes | Yes | Yes | Yes |
| Shan et al. 2018B | Yes | No | No | Yes | Yes | Yes | Yes | Yes | Yes | Yes |
| Sherlin et al. 2007 | Yes | Yes | Yes | Yes | Yes | Yes | Yes | Yes | Yes | Yes |
| Shungu et al. 2012 | Yes | U | Yes | N/A | N/A | Yes | Yes | Yes | N/A | Yes |
| Siessmeier et al. 2003 | Yes | No | Yes | Yes | Yes | Yes | Yes | Yes | Yes | No |
| Staud et al. 2018 | Yes | No | Yes | Yes | Yes | Yes | Yes | Yes | Yes | No |
| Tanaka et al. 2006 | Yes | Yes | Yes | Yes | Yes | Yes | Yes | Yes | Yes | Yes |
| van der Schaaf et al. 2017 | Yes | No | Yes | N/A | N/A | Yes | Yes | Yes | N/A | No |
| van der Schaaf et al. 2018 | Yes | No | Yes | Yes | Yes | No | No | Yes | Yes | No |
| Vuong et al. 2019 | Yes | Yes | Yes | N/A | N/A | Yes | Yes | Yes | N/A | Yes |
| Wu et al. 2016 | Yes | Yes | Yes | N/A | N/A | No | No | Yes | N/A | No |
| Yamamoto et al. 2004 | U | No | No | Yes | Yes | Yes | Yes | Yes | Yes | Yes |
| Yamamoto et al. 2012 | U | Yes | No | Yes | Yes | Yes | Yes | Yes | Yes | Yes |
| Zeineh et al. 2015 | Yes | No | Yes | N/A | N/A | Yes | Yes | Yes | N/A | Yes |
| Zinn et al. 2016 | Yes | Yes | No | N/A | N/A | Yes | Yes | Yes | N/A | Yes |
| Zinn et al. 2017 | U | Yes | No | N/A | N/A | Yes | Yes | Yes | N/A | No |
| Zinn et al. 2018 | Yes | No | Yes | N/A | N/A | Yes | Yes | Yes | N/A | Yes |

Supplementary Table: The Joanna Briggs Institute Checklist for Case Control Studies. Items answered as not applicable were removed from the final percentage. Abbreviations: JBI, Joanna Briggs Institute; Y, Yes; N, No; N/A, not applicable; U, unclear.

JBI Checklist items:

1. Were the groups comparable other than the presence of disease in cases or the absence of disease in controls?

2. Were cases and controls matched appropriately?

3. Were the same criteria used for identification of cases and controls?

4. Was exposure measured in a standard, valid and reliable way?

5. Was exposure measured in the same way for cases and controls? 6. Were confounding factors identified?

7. Were strategies to deal with confounding factors stated?

8. Were outcomes assessed in a standard, valid and reliable way for cases and controls?

9. Was the exposure period of interest long enough to be meaningful?

10. Was appropriate statistical analysis used?

**Justification**

**Armitage et al. 2009**

1. Healthy controls and participants are monozygotic twins therefore are age and sex-matched.
2. Recruited via Chronic Fatigue twin registry. Twins were required to be reared together and travel together to Seattle for the study.
3. All patients met the CDC Fukuda definition of ME/CFS. All controls didn’t.
4. No exposure. Study investigated power spectral analysis of sleep in twins discordant for ME/CFS.
5. As above.
6. Yes, confounding variables include intermitting sound and temperature. Adjustment of time zones.
7. These were controlled through use of temperature controlled, sound attenuated rooms. Participants were given a week to adjust to their set sleep schedule especially if travel to Seattle was required.
8. Measured using EEG
9. No exposure.
10. Statistical tests include MANOVA, T-tests and Chi square tests. Did not consider multiple comparisons.

**Barnden et al. 2011**

1. Healthy controls and participants were age-matched (within two years), sex-matched and weight-matched (within 5kg)
2. Recruited from community-based specialist and general practice (HCs from public) Location radius not provided.
3. Met both the Fukuda and Canadian criteria for CFS. All controls had no previous serious illness.
4. There was no exposure. Study used MRI and voxel- based morphometry to assess changes in autonomic structural integrity.
5. As above.
6. Confounding variables include: medications and existing pathologies.
7. These were controlled by ceasing all medications 2 weeks prior to the study and exclusion of participant data with detected abnormal pathological conditions that could potentially impact results. Those with bacterial and viral infections were given time to fully recover to ensure illness does not impact results.
8. Measured using MRI voxel-based morphometry.
9. There is no exposure period
10. Linear regression with adjustment for multiple regressions.

**Barnden et al. 2015**

1. Healthy controls and participants were age-matched (within two years), sex-matched and weight-matched (within 5kg)
2. Recruited from community-based specialist and general practice (HCs from public) Location radius not provided.
3. Met both the Fukuda and Canadian criteria for CFS. All controls had no previous serious illness.
4. There was no exposure. Study used MRI and voxel- based morphometry to assess changes in white matter.
5. As above.
6. Confounding variables include: medications and existing pathologies.
7. These were controlled by ceasing all medications 2 weeks prior to the study and exclusion of participant data with detected abnormal pathological conditions that could potentially impact results. Those with bacterial and viral infections were given time to fully recover to ensure illness does not impact results.
8. Measured using MRI voxel-based morphometry.
9. There is no exposure period.
10. Linear regression with adjustment for multiple regressions using false discovery rate (FDR).

**Barnden et al. 2016**

1. Healthy controls and participants were age-matched (within two years), sex-matched and weight-matched (within 5kg)
2. Recruited from community-based specialist and general practice (HCs from public) Location radius not provided.
3. Met the Canadian criteria for CFS. All controls had no previous serious illness.
4. There was no exposure. Study used MRI and voxel- based morphometry to assess autonomic changes in ME/CFS compared to HCs.
5. As above.
6. Confounding variables include: medications and existing pathologies.
7. These were controlled by ceasing all medications 2 weeks prior to the study and exclusion of participant data with detected abnormal pathological conditions that could potentially impact results. Those with bacterial and viral infections were given time to fully recover to ensure illness does not impact results.
8. Measured using MRI voxel-based morphometry.
9. There is no exposure period.
10. Linear regression with adjustment for multiple regressions using false discovery rate (FDR).

**Barnden et al. 2018**

1. ME/CFS patients and HCs were not matched.
2. Location radius not provided only provided where the scans took place.
3. Met the Fukuda criteria for CFS. Participants excluded if taking medication other than OCP/paracetamol, CFS didn’t meet full Fukuda inventory, BMI >35
4. There was no exposure. Study used T1 and T2 weighted spin echo scanning (MRI) to assess brainstem structures.
5. As above.
6. Confounding variables include: medications, not meeting full fukuda criteria, BMI.
7. Confounding factors were accounted for by exclusion.
8. Measured using T1 and T2 weighted spin echo scanning (MRI)
9. There is no exposure period.
10. P value was uncorrected voxel P<0.001. Did not adjust for multiple comparisons.

**Biswal et al. 2015**

1. Healthy controls and participants were age-matched. Whether it was sex-matched was not reported.
2. Population geographical data not recorded.
3. Met the Fukuda case definition for CFS. No definition of healthy volunteers.
4. There is no exposure. Study used Arterial spin labelling to measure regional and global absolute CBF.
5. As above.
6. Confounding variables not identified.
7. Controls for confounding variables not provided.
8. Measured using Arterial Spin Labelling.
9. There is no exposure period.
10. Individual parametric maps were generated, and a group statistical map was developed across all the healthy controls. Percent difference of ROIs between CFS patients and healthy controls were then calculated. A difference above a recognised threshold of P = 0.05. Did not adjust for multiple comparisons.

**Boissoneault et al. 2016**

1. Healthy controls and participants were age and sex-matched.
2. Recruited from University of Florida outpatient clinic – population geographical data not described.
3. ME/CFS patients were described according to the Fukuda case definition. Healthy controls were excluded if they presented with the following: chronic fatigue, chronic pain conditions, or mental illness.
4. No exposure. This study investigated resting state functional connectivity in ME/CFS patients compared to HCs using arterial spin-labelling fMRI.
5. As above.
6. Confounding factors include: heart disease, chronic obstructive pulmonary disease, malignancy, or other systemic disorders including psychiatric illnesses. Medications. caffeine, alcohol or psychoactive substances 24 hours prior to MRI.
7. Confounding factors were catered for by exclusion of participants that had a history of heart disease, chronic obstructive pulmonary disease, malignancy, or other systemic disorders including psychiatric illnesses. Those who took medications excluding vitamins were also excluded. Participants were instructed not to consume caffeine, alcohol or psychoactive substances 24 hours prior to MRI.
8. Yes, outcomes were measured using Arterial spin labelling (ASL) MRI
9. There is no exposure period.
10. Relationship between participants FC values and clinical and psychosocial factors was conducted using nonparametric (Spearman’s rho) correlation matrices. Consideration for normalisation, however, no mention of adjustment for multiple comparisons.

**Boissoneault et al. 2018**

1. Healthy controls and participants were age and sex-matched.
2. Geographical data not provided.
3. CFS participants met Centers for Disease Control criteria for chronic fatigue syndrome and were excluded from study if they reported a history of any other condition (including psychiatric illness) confounding CFS diagnosis. HC participants were excluded if they reported a history of chronic fatigue, chronic pain, or mental illness.
4. Fatigue induction protocol from PASAT
5. Measured and administered same way for both groups
6. Confounding factors include: history of any other condition confounding ME/CFS including psychiatrist illness. HC participants – history of chronic fatigue, chronic pain or mental illness. Caffeinated beverages, alcohol or psychoactive substances in the 24-hours prior to the study day. Medications except anti-hypertensives and/or vitamins.
7. Confounding factors were catered for by exclusion of participants that had a history of confounding disorders including psychiatric illnesses. Those who took medications excluding vitamins were also excluded. Participants were instructed not to consume caffeine (on the day of) or alcohol and psychoactive substances 24 hours prior to MRI.
8. Yes, outcomes were measured using Arterial spin labelling (ASL) MRI
9. Exposure was enough to induce fatigue that was statistically significant compared to baseline
10. Use of Pearson r correlation matrices on the basis of normalisation results. Did not consider multiple comparisons.

**Boissoneault et al. 2019**

1. Healthy controls and participants were age and sex-matched.
2. Population geographical data not recorded.
3. Patients were defined according to the Fukuda criteria. They were excluded based on confounding illness. This exclusion criteria also applied to HCs in addition to not having overlapping symptoms consistent with the Fukuda definition.
4. No exposure. Study investigated variability in cerebral blood flow (CBFV) and heart rate (HRV) in ME/CFS patients compared to HCs.
5. As above
6. Confounding factors identified including: multiple sclerosis, hypothyroidism, inflammatory muscle disease, or significant psychiatric illness (eg. Major depression or psychosis)
7. Confounding factors were catered for based on exclusion.
8. Yes, outcomes were measured using Arterial spin labelling (ASL) MRI
9. There is no exposure period
10. Paired T-test was used to determine significance between the HRV and fMRI sessions. Did not adjust for multiple comparisons.

**Caseras et al. 2006**

1. Age, sex- and handedness matched
2. Geographical data provided. CFS patients from king’s college London and St Bartholomew’s hospital and controls were members from the local community.
3. CFS participants met Centers for Disease Control criteria for chronic fatigue syndrome. Exclusion criteria for HCs were provided.
4. Exposure was *n- back task.* This task was consistent between each participant. All participants also received a training session prior to ensure they understood the task
5. Yes. All measurements were consistent between participants.
6. Confounding factors include: handedness, psychiatric disorder, history of brain injury, claustrophobia, suspected pregnancy, presence of any metallic implants and regular physical exercise.
7. Confounding factors were catered for by selecting only right-handed participants, exclusion of participants diagnosed with psychiatric disorder, history of brain injury, claustrophobia, suspected pregnancy, presence of metallic implants. Controls were limited to those who have low physical exercise.
8. Outcomes were measured using fMRI
9. A statistically significant outcome was found in this study with the given exposure period therefore it is enough.
10. Demographic data was measured using T tests. Gender and ground were calculated using chi square tests. Accuracy responding to the N back task number of correctly detected repetitions during each WM condition and the number of correctly identified X during control condition. As this dataset was not normally distributed a non-parametric test was selected. Type 1 error adjusted for.

**Chaudhuri et al. 2003**

1. Age and sex- matched.
2. No description of source population provided.
3. ME/CFS patients met the revised Fukuda criteria. No criteria was provided for HCs.
4. No exposure. Study investigated metabolic functions of the basal ganglia.
5. As above.
6. Medications that impact cerebral ^1^H MRI.
7. None were on medications that affect ^1^H MRI.
8. Yes, ^1^H MRI was used.
9. No exposure.
10. This study used a two- sample *t*-test with unequal variance to assess differences between the patient and control groups.

**Cleare et al. 2005**

1. No significant differences in any demographic information between ME/CFS and healthy controls. Handedness matched and timepoint matched.
2. Details on where ME/CFS patients were recruited from were detailed, however, no location details were provided for HCs.
3. Patients were included if they fulfilled CDC Fukuda criteria. HCs medical and psychiatric history were screened but was not clear as to whether participants were selected based on any exclusionary criteria. Therefore Unclear.
4. Yes, the exposure was [Carbonyl-11C] WAY-100635 and PET was acquired for 90mins post injection. A 10min transmission scan was also acquired.
5. No. The amount of activity was averaged out across participants, however, the amount received by ME/CFS patients was significantly higher than ME/CFS regardless of the moderate difference. All other demographic and PET related variables were not significantly different.
6. Confounding factors identified: medication, handedness, timepoints, past or current diagnosis of psychotic, melancholic or bipolar depression; psychosis; dementia; or eating disorder. Alcohol and substance abuse, underlying organic cause. Comorbid psychiatric disorder.
7. All patients underwent physical examination and relevant investigation, with a minimum of urinalysis, full blood count, urea and electrolytes, thyroid function tests, liver function tests, and erythrocyte sedimentation rate to rule out any comorbid condition. This also includes DSM-IV for assessing for a major psychiatric disorder.
8. Yes. Using Positron Emission Tomography and [11C] WAY-100635.
9. Decreased serotonin receptor number or affinity was observed using the 90 min timepoint therefore is enough time.
10. Repeated- measures ANOVA on regional BP and RI data. Used the Huynh- Feldt Epsilon to correct the observed *P* value.

**Cook et al. 2007**

1. Age and sex-matched.
2. Yes, recruited from the registry of CFS and HCs maintained by the CFS cooperative research centre at the University of Medicine and Dentistry of New Jersey.
3. Careful medical evaluation to determine suitability of ME/ CFS patients and HCs have been conducted. CFS patients were determined using the Fukuda case criteria. Individuals were excluded if they had the following: currently diagnosed heart or lung disease. If pregnant or breast feeding, had metal in any part of the body, had a severe head injury within the past 5 years or had current major depressive or generalized anxiety disorder. Exclusion based on medication classes.
4. Participants were exposed to a series of tasks including either an auditory monitoring or cognitive task. This was for a total duration of: physical activity, mood states and energy and fatigue traits were assessed.
5. Tasks were consistent for all participants including the time of given stimuli.
6. Confounding factors include: currently diagnosed heart or lung disease. If pregnant or breast feeding, had metal in any part of the body, had a severe head injury within the past 5 years or had current major depressive or generalized anxiety disorder. Exclusion based on medication classes.
7. Confounding factors identified were counteracted by exclusionary criteria in which participants were excluded if they had any of the confounding factors.
8. Yes. fMRI was used with focus on BOLD responses.
9. Significant results were found with the given time points.
10. False discovery rate correction with an overall p value of <0.05.

**De Lange et al. 2004**

1. Age, gender, sex and handedness matched.
2. Location radius not described.
3. All patients conformed to US Center for Disease Control and Prevention criteria for CFS (Fukuda et al., 1994). Subjects who manifested psychiatric comorbidity (e.g. depression) were excluded from the study. All participants assessed by detailed history and investigation, standardised psychiatric evaluation, computer assessment questionnaires, physical activity assessed by actometer during 2 weeks
4. Visual imagery and motor imagery tasks. Same number of stimuli in random order.
5. Tasks were consistent for all participants including the time of given stimuli.
6. Handedness, subjects who manifested psychiatric comorbidity (e.g. depression), gender.
7. Recruitment of only females, right-handed. Exclusion of subjects on the basis psychiatric comorbidity.
8. Yes, fMRI was used to measure ME/CFS patients’ performance on the motor or visual task.
9. Significant findings were found across all experiments with the given time points.
10. Regression with multiple comparisons - multiple regression was corrected for.

**De Lange et al. 2005**

1. Age, gender, sex and education matched.
2. Location radius not described.
3. All patients conformed to US Center for Disease Control and Prevention criteria for CFS (Fukuda et al., 1994). Subjects who manifested psychiatric comorbidity (e.g. depression) were excluded from the study. HC criteria not described.
4. No exposure. Study investigated cerebral morphology and volume in ME/CFS patients compared to HCs.
5. As above.
6. Those who took drugs that acted on the CNS or those who had a psychiatric comorbidity such as depression. Females vs. males also has some confounding factors.
7. Exclusion of those who had a psychiatric comorbidity or took drugs that acted on the CNS. Only females were included in this study.
8. Yes high resolution MRI images using voxel-based morphometry.
9. No exposure.
10. Global different in gray and white matter between groups were assessed with an ANCOVA. Regional differences in GM was assessed by T tends. Head size different were assessed with ANOVA and the correlation between daily physical activity and gray matter was assessed by means of Pearson’s correlation coefficients. Used family-wise error correction.

**Decker et al. 2009**

1. Subjects were Age, sex, BMI and race matched.
2. Participants were race matched and lived in Wichita in 1997
3. All patients conformed to US Center for Disease Control and Prevention criteria for CFS (Fukuda et al., 1994). Participated in the surveillance program. Did not exhibit any medical or psychiatric exclusion and had never reported fatigue of at least 1-month duration.
4. No exposure. Study investigated electroencephalographic correlates of ME/CFS compared to HCs using polysomnography.
5. As above.
6. Confounding factors identified include: age, sex, race, and body mass index in addition to significant confounding medical or psychiatric conditions.
7. These confounding factors were addressed through matching patients with two HCs that match in age, sex, race and BMI. Participants were also excluded based on significant confounding medical or psychiatric assessment.
8. Yes, polysomnography was used to measure outcomes which is used for many clinical symnographic observations.
9. No exposure.
10. Independent samples t-tests were used to assess values of alpha, delta, theta, beta and sigma between control subjects and ME/CFS subjects. This was measured at two-tailed significance of 0.05. Did not consider multiple comparisons.

**Finkelmeyer et al. 2018**

1. Subjects were age, sex, BMI and education matched.
2. Location radius provided: Newcastle and North Tyneside.
3. All patients conformed to US Center for Disease Control and Prevention criteria for CFS (Fukuda et al., 1994).
4. No exposure. Study investigated intracranial compliance and cerebral blood perfusion on ME/CFS patients compared to HCs.
5. As above.
6. Confounding factors identified include: positive for major depressive disorder or other psychiatric condition other than somatoform and anxiety disorders, use of medication that could affect the autonomic nervous system, pregnancy, substance abuse in the last 6 months, substance dependence in the last year, certain other medical conditions (diabetes) and contraindications for MRI. Age, sex, BMI and education were also identified as confounding factors.
7. Confounding factors were controlled by exclusion of these clinical cases. Participants were Age, sex, BMI and education matched to ensure these factors aren’t significantly contributing to the result observed.
8. Yes, MRI with arterial spin labelling was used.
9. No exposure.
10. Group comparisons were conducted using parametric test except for ASP which used a non-parametric rank test. To assess relationship among the measures Pearson bivariate and partial correlations were calculated. No adjustment for multiple comparisons.

**Finkelmeyer et al. 2018**

1. Subjects were age and sex-matched.
2. Location radius provided: Newcastle and North Tyneside.
3. All patients conformed to US Center for Disease Control and Prevention criteria for CFS (Fukuda et al., 1994).
4. No exposure. Study investigated regional grey and white matter differences in ME/CFS patients compared to HCs.
5. As above.
6. Confounding factors identified include: positive for major depressive disorder or other psychiatric condition other than somatoform and anxiety disorders or major physical health conditions.
7. Confounding factors were controlled by exclusion of these clinical cases. Participants were Age and sex to ensure these factors aren’t significantly contributing to the result observed. Intracranial volumes which was significantly higher in control participants compared to ME/CFS patients was accounted for through adjusting the calculation.
8. Yes, MRI with voxel-based morphometry was used.
9. No exposure.
10. Corrected for multiple comparisons.

**Flor- Henry. 2010**

1. Sex and dextral matched but not age-matched.
2. No location radius provided.
3. All satisfied Fukuda's criteria (1994), all were dextral and were unmedicated at the time of study. For HCs, any who responded in the affirmative to having a history of neurological disease, birth complications, head trauma, psychiatric illness or substance abuse were excluded from the study.
4. EEGs were recorded from all subjects during a passive condition, eyes open (EO) and two active cognitive conditions: word finding and dot localisation. To ensure consistency – subjects answered in 5s but the 1^st^ and last second of the EEG recording was not counted. To ensure compliance, only those EEGs corresponding to correct responses to the test questions were utilised.
5. Tasks were consistent and at least 3mins of recording was obtained for each participant. Given to the participant in the same way (slides projected from the rear of the recording chamber. The offset of the bottom rectangle differed randomly among items such that half of the items were offset to either the right or the left side.
6. Confounding factors identified: skull thickness.
7. Each matrix was then normalized by dividing all of it elements by its trace (the sum of its diagonal elements) to account for skull thickness.
8. Yes, EEG was used. Used previously published cognitive and motor activities that effectively was able to show the outcome.
9. Statistically significant results were observed in the time allocated.
10. The elements of these vectors from ME/CFS were statistically compared to identify brain regions that had statistically significant differences. This used randomized T tests (Holmes value). This method does not rely on distribution assumptions and reduces the probability of obtaining false-positive results. Significance is denoted by P < 0.05 (two sided)

**Gay et al, 2016**

1. Sex and age matched.
2. Outpatient clinics at the University of Florida for ME/CFS patients and local advertising for healthy controls.
3. Fulfilled 1994 Case definition and 2003 CCC for ME/CFS. HCs were defined as: Subjects that did not have fatigue symptoms. Subjects with a history of heart disease, COPD, malignancy, or other systemic disorders were excluded, including those with psychiatric illnesses. (Reeves et al., 2003).
4. No exposure. This study investigates resting-state functional connectivity in ME/CFS patients and HCs.
5. As above.
6. Confounding factors were not identified.
7. Did not detail response to confounding factors.
8. Yes, fMRI was used – this was assessed using two methods data driven and model based.
9. No exposure.
10. T statistic was calculated for both methods. This result was corrected for multiple comparisons using alphasim for a voxel <0.001.

**Kim et al. 2015**

1. Age, handedness and sex-matched.
2. All were recruited from local community through flyers, announcements or word of mouth – Seoul, South Korea.
3. All satisfied revised diagnostic criteria of CFS (Fukuda 1994). All participants underwent clinical interviews to exclude those with other psychiatric illnesses, then administered structured interviews by a clinical research psychologist to assess functional status and symptoms
4. No exposure. Study investigated altered resting-state functional connectivity in women with ME/CFS compared to HCs.
5. As above
6. Confounding factors identified: Current or past psychiatric disorders, traumatic brain injury, neurological illness, relevant visual defects or any radiological contraindications for MRI scanning.
7. Participants with the following confounding factors were excluded from the study.
8. Yes fMRI was used.
9. No exposure
10. Independent T- tests between HCs and CFS patients were performed with age and self-report scales including the Chalder Fatigue Scale, BDI, BAI and PGWBI. Partial correlation analysis tested relations between the functional seed-target connectivity and clinical scale scores. Statistical analyses were conducted on SPSS with p < 0.05, two tailed. Did not correct for multiple comparisons.

**Lange et al. 2005**

1. For study 1: age and sex-matched for study 2: sex but not age-matched. All were also right-handed
2. No location radius provided.
3. All patients fulfilled the 1994 Fukuda case definition. Standard intake included careful medical evaluation to determine group membership, computerised structured psychiatric interview, comprehensive neuropsychological testing battery, PASAT for verbal information processing.
4. Two experiments with two different exposures. Task 1. Auditory monitoring Task 2. mPASAT verbal task. Timing and delivery was consistent between all participants.
5. Yes, output for this was measured using fMRI.
6. Confounding factors identified: handedness, intellectual ability and medications.
7. All participants were right-handed, had am estimated premorbid intellectual ability within the average range as assessed by participants’ Standard Score on the Vocabulary subtest of the WAIS-R obtained through standard intake procedure. Participants were only allowed to take birth control pills.
8. Yes, fMRI was used.
9. Timing provided was sufficient to obtain results.
10. ROI analysis was employed. All within group ROI results were then corrected for multiple voxel-wise comparisons with the False Discovery Rate (FDR method) and analysed at the corrected P level of <0.05.

**Le Bon et al. 2012**

1. Exact match on gender and within 2-year age difference between ME/CFS patients and HCs.
2. Location radius not clear. Referred to the sleep unit of the Brugmann University Hospital by the medical check-up. Locally recruited HCs.
3. CDC criteria were used for a first selection of CFS patients. For HCs no significant somatic condition and no current or past mental disorder were allowed. Further exclusion criteria were identical to patient groups.
4. No exposure. Study investigates ultra-slow delta power using EEG in ME/CFS patients and HCs.
5. As above.
6. Confounding factors identified: unauthorised sleep- wake schedules. Alcohol consumption (greater than 2 units per day) Caffeine-including beverages.
7. This was monitored using sleep diaries, but no participants needed to be excluded on this basis. Those who had alcohol consumption greater than 2 units per day were excluded. Caffeine beverages were not allowed post 3pm.
8. Yes, EEG was used to measure delta activity in participants.
9. No exposure.
10. Independent samples t-tests were used to assess values of Ultra-Slow, Delta, Theta, Alpha, Sigma and Beta between CFS patients and HCs. Effect size was measured using Cohen’s D. Group differences were considered significant at two-tailed significance of 0.05. Did not adjust for multiple comparisons.

**Lewis et al. 2001**

1. Age, sex, BMI zygosity-matched twin study.
2. Twins reared together.
3. Twin with ME/CFS met CDC criteria for CFS (Fukuda). The control twin was healthy through reviewing subjects’ medical chart to verify the illness and health status of the twin. Must be reared together.
4. Exposure was 30 mCi (1,110 MBq) of technetium 99m (99mTc) hexamethylpropyleneaHMPAO) a radioactive tracer that localizes in the brain in accordance with rCBF, this was administered intravenously to both twins simultaneously to allow exposure to be provided in a standard manner.
5. Yes, the radiolabelled tracer was measured using SPECT for both patients and controls.
6. Confounding factors include: being reared separately, alcohol, caffeine and medications that affect cognition or sleep.
7. Confounding factors were addressed by requesting participants to cease the consumption of alcohol, caffeine and medications that affect cognition or sleep two weeks prior to the study commencing. Those who were unable to were excluded from the study.
8. Yes, SPECT was used.
9. Exposure time was enough according to clinical guidelines. This is also clarified by results being observed in the given time.
10. Did not adjust for multiple comparisons.

**Mathew et al. 2008**

1. ME/CFS patients and HCs were matched for age, sex, body mass index, handedness and IQ.
2. Source population information was provided for ME/CFS patients but not HCs.
3. ME/CFS patients were diagnosed according to the modified CDC Fukuda criteria. HCs were medically healthy and did not have any current medical conditions or axis I psychiatric disorders.
4. No exposure. Study investigated ventricular cerebrospinal fluid lactate levels in ME/CFS patients compared to HCs.
5. As above.
6. Alcohol and medications. Differences In ventricular size and partial- volume averaging.
7. Adjustments were made for differences in ventricular size and partial-volume averaging through tissue tissue- segmented and co-registered volumetric MRI data. All medications and alcohol were ceased 48 hours prior to the procedure.
8. Yes, ^1^H MRSI was used.
9. No exposure.
10. Post hoc analyses were performed using the Tukey HSD test and non-parametric Mann-Whitney tests.

**Mueller et al. 2019**

1. Age and sex- matched
2. Source population information not provided.
3. ME/CFS patients were diagnosed according to the Fukuda case definition of ME/CFS. HCs had an average self-report daily fatigue rating of <2.
4. No exposure. Study investigated brain metabolites in ME/CFS patients compared to HCs.
5. As above.
6. MRI safety contraindications, psychostimulant or opioid use. Smoking or use of anti-inflammatory drugs.
7. Participants were requested to stop the use of NSAIDS 24 hours prior to the procedure. Those who had MRI safety contraindications, psychostimulant or opioid use were excluded from the study.
8. Yes, MRS was used.
9. No exposure.
10. Main analyses used univariate independent *T*-tests. For the number of regions of interest tests an additional false discovery rate of 0.01 was used.

**Murrough et al. 2009**

1. ME/CFS patients were not age or sex-matched.
2. Did not have significant differences in source population characteristics.
3. ME/CFS patients were diagnosed according to the US CDC guidelines. HCs did not meet criteria for ME/CFS and were free from psychiatric disorder.
4. No exposure. Study investigated ventricular lactate levels in ME/CFS patients compared to HCs.
5. As above.
6. Confounding factors include: psychotropic medication
7. Participants were psychotropic medication free for between two to four weeks depending on medication type.
8. Yes ^1^H MRS was used.
9. No exposure.
10. One way ANOVA and was used for continuous variables or Chi square for dichotomous variables. Post hox analyses were performed using the Tukey’s honest significant difference (HSD).

**Nakatomi et al. 2014**

1. Age, handedness (all right handed) and sex-matched.
2. Patients recruited from the Fatigue Clinical Center at Osaka City University Hospital, Osaka, Japan. No location data is provided regarding the HCs.
3. Patients diagnosed according to the International diagnostic criteria (CDC, Fukuda). HC’s had no symptoms related to fatigue and no problems in their daily activities.
4. 11C-(R)-PK11195 exposure. Injected 30sec after the start of the PET scan. Traver dose and specific activity was consistent across all participants.
5. Yes, it was measured using PET.
6. Medications that affect the autonomic nerve function including: beta blockers, benzodiazepines, corticosteroids, and medications known to affect the CNS (eg: methylphenidate, dexamphetamine, antidepressants, and antipsychotic drugs.
7. Potential participants who took those medications were excluded from the study.
8. Yes, PET was used.
9. Time was enough as there were results of significance in the given time.
10. Symptom severity, blood-cytokine data, and regional 11C-(R)-PK11195 BPND values were compared across the groups using Student t tests. Categoric variables were compared across the groups using Fisher exact tests. The relation between symptom severity scores and 11C-(R)-PK11195 BPND values was analysed using Pearson correlation in All P values were 2-tailed, and values of less than 0.05 were statistically significant. Correction for multiple comparison were made.

**Neu et al. 2011**

1. Age, handedness and sex- matched (female only)
2. No location radius was provided.
3. Full medical check-up was conducted on all participants. CFS/ME patients were diagnosed according to the CDC Fukuda criteria. Final inclusion was determined through polysomnography to exclude diagnosis with primary sleep disorder. Control subjects had no significant somatic conditions and no current or past mental disorders. Further exclusion criteria were identical
4. No exposure. Study investigated cognitive impairments in CFS/ME compared to HCs.
5. As above.
6. Shift work, day time napping, neuropsychopharmacological treatment (including pain medication).
7. Daytime napping was not allowed nor was shift working. Neuropsychopharmacological treatment had to be ceased 2 weeks prior to the study commencement date.
8. Yes. EEG was used.
9. No exposure.
10. Parametric tests were used for each variable. Between- group comparisons involving continuous data. These were computed using MANOVA and single factor (subject group). Hypotheses tests were two-sided and carried out at a 5% significance level. Did not adjust for multiple comparisons.

**Neu et al. 2014**

1. CFS patients and controls show no difference in age, sex or BMI.
2. No location radius was provided.
3. ME/CFS participants were defined according to CDC Fukuda criteria. No clear criteria were provided for HCs.
4. No exposure. Study investigates non-REM sleep EEG power distribution in CFS/ME patients compared to HCs.
5. As above.
6. Confounding factors include: hypnotics or other relevant neuropsychopharmacological (including antidepressants) and sleeping time
7. Cease neuropsychopharmacological (including antidepressants) for at least 2 weeks prior to recording. Participants required a minimum time in bed (TIB) threshold of 300min and a minimum total sleep time (TST) threshold of 180min.
8. Yes, EEG was used.
9. No exposure.
10. Hypothesis tests were performed two-sided at the 5% significance level. A Kolmogorov- Smirnov test was used to assess normality for continuous variables. Non-normal distributed data was rank converted. Between group comparisons were assessed using MANCOVA. Posteriori tests were performed using a sequential Bonferroni (Dunn–Sidak) correction for multiple comparisons.

**Okada et al. 2004**

1. Participants were age-matched. Unclear whether ratios of male-to- female were sex-matched.
2. Participants recruited from the outpatient fatigue clinic in Osaka University Hospital.
3. ME/CFS participants were defined according to the CDC Fukuda criteria. While controls were described as healthy no detailed criteria was provided.
4. No exposure. Study investigated gray-matter volume in ME/CFS and HCs.
5. As above.
6. Potential confounding effects include age, sex and whole segment (gray or white matter) volume differences
7. These factors were modelled and the variances attributed to them were excluded from the analysis.
8. Yes. Voxel- based morphometry MRI was used.
9. No exposure.
10. The significance levels for statistics estimated by 500 nonparametric randomization and permutation tests were set at P = 0.05, corrected for multiple comparisons. Within the areas showing a significant volume reduction in patients, linear correlates between volume reduction and the degree of fatigue were examined under the threshold of P < 0.005.

**Puri et al. 2002**

1. Age, handedness, education, socioeconomic status and sex- matched.
2. No source information was provided.
3. Patients met Fukuda et al. HC had no known medical or psychiatric disorder.
4. No exposure. Study investigated metabolite levels in ME/CFS patients compared to HCs.
5. As above.
6. No confounding factors identified.
7. As above.
8. Yes MRS was used.
9. No exposure.
10. No statistical analysis methods described.

**Puri et al. 2012**

1. The mean age of the patients and the male-to-female ratio of the participants did not differ significantly from the patients to the HCs.
2. No location radius was provided.
3. Patients met revised criteria for CFS of the CDC Fukuda criteria. None of the healthy controls met the CFS criteria nor did they suffer from undue fatigue or from any history of neurological or psychiatric disorder.
4. No exposure. Study investigated regional grey and white matter volumetric changes in ME using voxel-based morphometry 3T MRI study.
5. As above.
6. Confounding factors not described.
7. Addressing of confounding factors not described.
8. Yes outcomes were measured using Voxel based MRI.
9. No exposure.
10. Voxel-wise generalised linear modelling was applied using permutation-based non-parametric testing, forming clusters at t>2.3 and testing clusters for significance at p< 0.05, corrected for multiple comparisons across space.

**Schmaling et al. 2003.**

1. Gender, age and education matched.

2. participants were matched in ethnicity.

3. ME/CFS patients met Fukuda definition. Potential healthy participants who reported chronic fatigue, met criteria for CFS or fibromyalgia. Had a major medical illness (such as high blood pressure, diabetes, or severe asthma), or any current or historic psychiatric diagnosis (including alcohol or drug abuse) were excluded.

4. Exposure was 30mCi of 99mTc-ECD radiotracer and PASAT test. PASAT involves listening to an audiotape and presents s random sequence of single digits. The subject adds the last two numbers

5. Yes, SPECT and PASAT was used. Consistent across all participants.

6.medications that affect cerebral functioning, previous neuropsychological testing, left handedness, history of fever for several hours or longer, severe claustrophobia, learning disability or special education program, or exposure to radiation above two rads in the last year.

7. exclusion of all participants with characteristics listed above.

8. Yes SPECT was used.

9. Timing of exposure was sufficient to achieve significant results.

10.Used ANOVA (MANOVA, ANCOVA), did adjustments for covariates. Did not adjust for multiple comparisons.

**Shan et al. 2016**

1. Subjects were age- matched. Unclear if sex-matched.

2. No location data provided.

3. ME/CFS patients met both Fukuda and Canadian criteria. NCs were not taking any medications and had no previous serious illness.

4. No exposure. Study investigated progressive brain changes in patients and HCs using MRI.

5. No exposure.

6. Confounding factors include: anti-depressants and handedness

7. Those who took antidepressants were requested to stop them for 1 week before their MRI study. All participants were right-handed.

8. Yes MRI was used.

9. No exposure.

10. Paired two tailed t-tests were used. Bonferroni correction for multiple comparisons of the significant differences from zero. Corrected to P< 0.0071 statistical significance.

**Shan et al. 2017**

1. There were no significant differences in age and all participants were handedness matched. Patient and control groups were not sex matched.
2. No location radius provided.
3. Patients met Canadian Consensus Criteria. Normal controls has developed a significant medical condition including hypertension, or psychiatric illness during research period. No subject was taking centrally acting medications, non-smokers and not substance abusers.
4. There was no exposure. Study investigated unrefreshing sleep and brain structure variations using MRI spin echo.
5. As above.
6. Confounding factors included: smoking, substance abuse, medical condition, handedness, psychiatric illness.
7. Confounding factors were controlled for by excluding participants that were smokers, substance abusers, had confounding medical conditions or psychiatric illness. All participants were handedness matched and were right handed.
8. Yes, outcomes were measured using MRI – spin echo.
9. No exposure.
10. Outcomes were statistically assessed using independent two sample two tailed t test to assess and compare intergroup differences in clinical measures, global GM values and global WM volumes. Bonferroni correction was applied to counteract multiple comparisons of six characteristics. Pearson correlation between each clinical measure and global GM and WM volumes from all subjects were calculated. Bivariate tests of significant differences in correlation coefficient from zero with two tzaisl were followed with Bonferroni correction for multiple comparisons of 15 pairs to test for uncorrected (P <0.05) and corrected (P < 0.003) statistical significance.

**Shan et al. 2018**

1. Participants were age matched. Recruited to match female/ male ratio.
2. No location radius was provided.
3. Patients met Fukuda criteria. All right-handed, non-smokers, not substance abusers.
4. Exposure: stroop task. Randomized event-related colour word variant during fMRI acquisition. Same session time 40% congruent and 30% incongruent. 30% neutral. Stroop effect was accounted for.
5. Exposure was randomized between all groups
6. Confounding factors included: CNS medication or ones that cause cerebral hemodynamic responses or subjects which did not meet the full fukuda criteria.
7. Participants with the following confounding factors were excluded from the study.
8. Yes, outcomes were measured using MRI.
9. There were significant findings in the given time therefore it was sufficient.
10. Differences between CFS and NC groups in clinical variables and in the SampENs were compared using the independent-samples t-test. The Pearson correlation was used to determine correlations between clinical variables and SampEns across all subjects followed by a conversion to FDR-Q values.

**Shan et al, 2018**

1. Participants were age matched. Recruited to match female/ male ratio.
2. No location radius was provided.
3. Patients met Fukuda criteria. No information was provided on HCs.
4. Exposure: stroop task. Randomized event-related colour word variant during fMRI acquisition. Same session time 40% congruent and 30% incongruent. 30% neutral. Stroop effect was accounted for.
5. Exposure was randomized between all groups
6. Confounding factors included: CNS medication or ones that cause cerebral hemodynamic responses or subjects which did not meet the full fukuda criteria.
7. Participants with the following confounding factors were excluded from the study.
8. Yes, outcomes were measured using MRI.
9. There were significant findings in the given time therefore it was sufficient.
10. Pearson correlation was used to determine correlation (two tailed p<0.05) between MRI measures and signal SampEns, FCs and DFC SampEns and disease severity.

**Staud et al. 2018**

- - - 1. Participants were age and sex-matched.
      2. University of Florida outpatients but no information on advertising patients.
      3. All ME/CFS subjects met the 1994 CDC Criteria and the 2003 Canadian Criteria for ME/CFS. ME/CFS subjects could not have a history of heart disease, systemic sclerosis, malignancy, or other systemic disorders including psychiatric illnesses that would be exclusionary for a diagnosis of ME/CFS. No medications except vitamins. They were excluded if they had a previous history of chronic fatigue, depression or cancer.
      4. rCBF was measured at rest and during a strenuous exercise.
      5. This was measured using arterial spin labelling BOLD fMRI.
      6. Confounding factors include: Caffeine and medications
      7. No caffeine intake was allowed for at least 12h prior to the study.
      8. Yes, outcomes were measured using BOLD fMRI.
      9. The given exposure time provided significant findings therefore is enough.
      10. Analysis of covariance (ANCOVA) was used to identify voxels within the a *priori ROI* mask during both fatigue induction and recovery. Differences between ME/CFS and HC in the correlation between rCBF and fatigue were tested by comparing Fisher’s *r-to-z* transformed correlation coefficients. Potential differences in demographic and affective variables between ME/CFS and HC were assessed using independent *t-tests.* Group and time affects on in-scanner fatigue ratings were assessed using rmANOVA. Did not adjust for multiple comparisons.

**Miller et al 2014.**

Age, sex, Ethnic, BMI and education matched.

Ethnic matched. Location radius provided: Georgia.

CFS cases were determined using the 1994 case definition. Medical and psychological exclusions for CFS include history of head trauma or seizures; unstable cardiovascular, endocrine, hematologic, renal or neurologic disease (determined by physical examination and laboratory testing); hepatitis B or C or human immunodeficiency virus infection (by medical history); current or history of schizophrenia or bipolar disorder or a current diagnosis of major depression [as determined by Structured Clinical Interview for Diagnostic and Statistical Manual of Mental Disorders–Fourth Edition (SCID)][24]. Subjects with anxiety disorders were not excluded. Non-fatigued controls.

Participants are exposed to a previously published gambling task proven to elicit activation of basal ganglia structures. Valid because it is proven effective based on previous published research. The game had the same outcome as controlled by the gamemaker. “This procedure allowed for experimental control of the task while masking the deterministic nature of the game from the participant – realistic.

Measured using MRI across all participants in the study.

Yes, psychotropic medications including antidepressant, antipsychotic, mood stabilizer medications. Alcohol/ psychoactive substance abuse or dependence.

All subjects were free of psychotropic medication for 4 weeks prior to the study. Those who were dependent or abusing alcohol or psychoactive substances within the past year were excluded. Study was age, sex, ethnic, BMI and education matched.

Yes outcomes were measured using fMRI.

Time was enough based on previously published data. Results were gathered in the given time additionally therefore clarifying it is enough.

Did choose appropriate statistical test unequal variances. Did not correct for multiple comparisons.

**Seissmeier et al. 2003.**

1. Patients and controls were age and sex-matched.

2. Patients assessed in the University Hospital in Mainz. Referred to a normal database but no information was provided regarding normal location radius.

3. Patients fulfilled the CDC diagnostic criteria for CFS and the 1992 CDC working case division. Normal controls were neurological or psychiatric disease free this was assessed by physical examination and extensive neuropsychological testing (SCID)

4. Exposure is 18-fluorodeoxyglucose (FDG). All studies were done under identical standardised resting conditions with closed eyes and in dim ambient light.

5. This was measured using PET.

6. Confounding factors include: dead time, scatter and random coincidence.

7. These factors were corrected for when developing the images.

8. Yes PET was used.

9. The time was enough to show a response in some of the ME/CFS sample.

10. Uncorrected for multiple comparisons.

**Shan et al. 2018**

1. Participants were age matched. Recruited to match female/ male ratio.
2. No location radius was provided.
3. Patients met Fukuda criteria. No criteria was defined a part from matching age and female-to-male ratio.
4. Exposure: stroop task. Randomized event-related colour word variant during fMRI acquisition. Same session time 40% congruent and 30% incongruent. 30% neutral. Stroop effect was accounted for.
5. Exposure was randomized between all groups
6. Confounding factors included: CNS medication or ones that cause cerebral hemodynamic responses or subjects which did not meet the full fukuda criteria.
7. Participants with the following confounding factors were excluded from the study.
8. Yes, outcomes were measured using MRI.
9. There were significant findings in the given time therefore it was sufficient.
10. Differences between CFS and NC groups in clinical variables and in the SampENs were compared using the independent-samples t-test. The Pearson correlation was used to determine correlations between clinical variables and SampEns across all subjects followed by a conversion to FDR-Q values. The variables that were significantly correlated with health scores were further analysed using a hierarchical regression analysis to determine if those measures contributed to the variance in the health scores across all subjects.

**Sherlin et al. 2007**

- - - 1. CFS and HCs were age and sex matched (monozygotic twins).
      2. Twins were reared together.
      3. Discordant twin for ME/CFS was assessed according to Fukuda criteria. Additionally, they were medically assessed for exclusionary conditions. Non-fatigued twin complied to the following criteria: was healthy and not fatigued.
      4. There were three different conditions eyes open, eyes closed, and eyes closed while performing a mental mathematics task. All were required to perform the task and artifects including eye blinds, eye movements, teeth clenching, body movements or EKG artefact were removed. Therefore, allowing the data to be an appropriate representation.
      5. Yes, the brain activity is measured using EEG LORETA.
      6. Confounding factors include: eye blinks, eye movements, teeth clenching, body movements or EKG artefacts –
      7. Artefacts were manually removed.
      8. Yes, EEG LORETA
      9. Significant results were observed in the given time, hence, shows time is sufficient.
      10. Voxel by voxel T-tests were computed.

**Shungu et al. 2012**

1. Age and sex matched to ME/CFS patients.

2. HV’s were recruited through local media advertisement. CFS patients were recruited via clinician referrals and media advertisements. Unclear of location radius of CFS patients.

3. Patients were diagnosed according to the modified US Centers for Disease Control and Prevention (CDC). HVs did not meet the criteria for ME/CFS or any psychiatric disorder.

4. No exposure. Study investigates the relationship between cortical glutathione and clinical symptoms in ME/CFS patients compared to HCs.

5. As above

6. Lactate CH3 group has nearly the same MR frequency as the ethanol 1.3ppm

7. The participants stopped drinking alcohol at least 48 hours prior to the study commencing.

8. Yes, MRSI and structural MRI were used.

9. No exposure.

10. Normality of data was assessed using the Shapiro- Wilks test. Type 1 error rate was protected against by limiting the statistical tests only to out specified hypotheses. Bonferroni correction was applied for multiple comparisons. For all normally distributed secondary outcome measures three-group comparisons were performed using general linear models (ANOVA) followed by post-hoc comparisons with Tukey’s honestly significant difference test. two-tailed, with the level of significance set at a<0.05,

**Tanaka et al. 2006**

Age and sex- matched (all male)

Outpatients of the Department of Hematology and Oncology of Osaka University.

Patients were classified according to the Fukuda criteria. HCs had no history of psychiatric or neurological illness among the normal subjects. None of the normal or CFS subjects were taking medication known to affect cerebral blood flow, all had normal auditory function, and they were all right-handed according to the Edinburgh handedness inventory.

all subjects completed visual search tasks 3 times, for 6 min each, to evaluate their capacity to perform this task. Number of targets was selected based on performance during pre-imaging,

This was measured using fMRI.

Confounding factors: difference in task difficulty among the subjects.

Task difficulty was set based on the performance on the visual task during the trials.

Yes, fMRI.

Significant differences in responses were observed in the given time.

The threshold for the SPM{Z} of individual analyses was set at P < 0.05 with a correction for multiple comparisons at the cluster level of the entire brain.

**Van der Schaaf et al. 2016**

1. CFS and HCs were age, sex, education and BMI matched.

**2.** No location information provided.

3. CFS patients met CDC Fukuda criteria for diagnosis. Additional inclusion criteria for HCs were a score ,35 on the CIS-fatigue subscale and no chronic medical condition, including no chronic pain.

4. No exposure. Study measures gray matter volume (GMV) and the N-acetylaspartate and N-acetylaspartylglutamate/creatine ratio (NAA/Cr) and its relation to prefrontal structure in ME/CFS patients and HCs.

1. As above.
2. Confounding factors: BMI, psychotropic medications, psychiatric disorders contraindication for MR examinations, poor hearing and vision and insufficient command for Dutch language.
3. no use of psychotropic medications 6 months before testing, no contraindication for MR examinations, normal hearing and vision and enough command for dutch language.
4. Yes, outcomes were measured using MRI.
5. No exposure.
6. Two-sided independent sample *t*-tests were performed for group characteristics, clinical measures, psychomotor speed, activity level, DLPFC GMV, global GMV and NAA/Cr. No adjustment for multiple comparisons.

**Van der Schaaf et al. 2018**

1. Gender, age and education matched.

2. No demographic information was provided.

3. ME/CFS patients met Fukuda definition. HCs had <35 on CIS-fatigue and no chronic medical condition

4. Exposure was feedback processing and motor preparation behavioural task (physical force production task)

5. Yes, fMRI and a physical force production task was used. Consistent across all participants.

6. No confounding factors identified.

7. As above.

8. Yes fMRI was used.

9. Timing of exposure was sufficient to achieve significant results.

10. statistical inferences are based on alpha-level of 0.05 there were family-wise error correction made for multiple comparisons. Name of tests were not provided?

**Vuong et al. 2019**

1. Subjects were age and sex matched

2. Location radius provided: Newcastle and North Tyneside.

3. Patients conformed to US Center for Disease Control and Prevention criteria for CFS (Fukuda et al., 1994). HCs had no diagnosis of ME/CFS, screened negative for TMD and had no other history of major psychiatric and physical health conditions.

4. No exposure. Study investigated whole brain autonomic processes in ME/CFS patients compared to HCs.

5. As above.

6. Confounding factors identified include: positive for major depressive disorder or other psychiatric condition other than somatoform and anxiety disorders, use of medication that could affect the autonomic nervous system, pregnancy, substance abuse in the last 6 months, substance dependence in the last year, certain other medical conditions (diabetes) and contraindications for MRI. Age, sex, BMI and education were also identified as confounding factors.

7. Confounding factors were controlled by exclusion of these clinical cases. Participants were Age, sex, BMI and education matched to ensure these factors aren’t significantly contributing to the result observed.

8. Yes, fMRI was used.

9. No exposure.

10. F tests, whole-brain pairwise pairwise T-tests. P (uncorrected) <0.05.

**Wu et al. 2016**

1. Age and Sex-matched between patient group and controls.
2. Han nationality outpatients, male and female, of the Subsidiary Hospital of Shandong University of traditional Chinese Medicine were selected.
3. Patients met Fukuda criteria along with exclusionary criteria: anxiety and depression. Patients with severe primary diseases, including cardiovascular and cerebrovascular diseases, pneumonopathy, hepatopathy, nephropathy and hematopoietic system diseases were also excluded. Control group is exclusionary criteria + no physiologic diseases or psychological disorders and did not meet the earlier diagnostic criteria of CFS at the same time.
4. No exposure. Study investigates characteristics of patients with ME/CFS using routine EEG.
5. As above.
6. No confounding factors detailed
7. As above.
8. Yes, clinical EEG was used.
9. No exposure.
10. Data were expressed as mean ± standard deviation. Unpaired Student’s t-test was used to compare the difference between two groups. P,0.05 was considered statistically significant. Did not detail whether additional measures were implemented to control for type 1/ type 2 error.

**Yamamoto et al. 2004**

Age matched, unclear if sex-matched.

Location data not provided.

Patients were characterised according to the CDC Fukuda criteria. Healthy control criteria was not described.

A radiotracer [11C] (+)McN5652 was used to measure density of serotonin transporters.

This was measured using PET.

Confounding factors: patients with a major depressive disorder determined by diagnostic and statistical manual of mental disorders and taking drugs affecting 5-HT neurons.

Participants that took drugs that acted on/ affected 5-HT neurons within 1 month prior to the start of the study were excluded.

Yes, MRI and PET were used.

Significant results were observed in the given time therefore this is sufficient.

Two-tailed *t*- tests between patients and controls and conducted correlation analysis between the BP value and the clinical symptomatic scores of patients. Significance was corrected at a score of P<0.05 for the two tailed tests. For correlation analysis significance was measured at *p <0.001.*

**Yamamoto et al. 2012**

**1.** Unclear if participants were age or sex-matched.

**2.** All study participants were Asian.

1. ME/CFS patients classified according to the clinical diagnostic criteria (Fukuda), however, HC criteria are not detailed.
2. Yes, participants were exposed to different tests including one that predicts IQ, measurements of executive functions (through a card sorting task), another that measures mental fatigue through advanced trail-making, non-verbal long-term memory and memory function tests. These all follow protocol of previously conducted experiments.
3. Yes PET was used to measure these exposures.
4. Confounding factors include: smoking, drinking alcohol regularly and taking medications known to affect the central cholinergic system including AChE inhibitors. All participants were medicated with vitamin C and the Chinese herbal medicine hocheukkito (but no study suggests impact on mAChR.
5. To account for these confounding factors those who smoke, drink alcohol regularly and take medications known to affect the central cholinergic system including AChE inhibitors were excluded.
6. Yes, MRI and PET were used.
7. Exposure time was sufficient in obtaining results.
8. Statistical significance was set at P< 0.05. Age, extent of fatigue, results of neuropsychological tests and regional BPND values or uptake were compared among 3 groups with one way ANOVA using a post hoc Student-Newman-Keuls test.

**Zeineh et al. 2015**

1. ME/CFS and HCs were age (within one year) and sex matched.
2. No location radius provided.
3. Patients met at least 4 of the eight Fukuda symptoms: impaired memory or concentration, sore throat, tender joint pain, unrefreshing sleep, and post exertional malaise. Healthy controls had no history of major depression, CFS or Chronic Fatigue or substance abuse in the past year.
4. No exposure. Gray and white matter and cortical thickness were assessed using ASL.
5. As above.
6. Handedness.
7. Participants were grouped based on handedness. Left-handed and ambidextrous participants were pooled into non-right-handed group.
8. Yes, outcomes were measured using ASL.
9. No exposure.
10. Multiple statistical tests were used. Bonferroni correction applied and correction for multiple comparisons.

**Zinn et al. 2016**

1. Unclear if age/ sex matched (given as a grouped average). Ethnicity matched and handedness matched.
2. All participants were ethnicity matched and visited De Paul University to have their EEG recorded.
3. All satisfied Fukuda's criteria (1994) or CCC. HC criteria has not been detailed.
4. No exposure. Study used eLORETA to assess current source density and functional connectivity.
5. As above.
6. Confounding factors: handedness, medications.
7. No participants were taking medications that would affect the EEG. Participants all had the same handedness.
8. Yes, eLORETA was used.
9. No exposure.
10. Voxel-by-voxel independent sample F-ratio tests were used to evaluate the differences based on eLORETA Controlling of type 1 error occurred through adjusting for multiple comparisons.

**Zinn et al. 2017**

1. Participants matched by sex but not age. Age has been statistically adjusted, however to account for this difference as it has a significant.
2. All participants visited DePaul university to have their EEG recorded.
3. All satisfied Fukuda's criteria (1994) or CCC. HC criteria has not been detailed.
4. No exposure. This study assessed the relationship between complex brain networks and neurocognitive impairment in CFS patients and healthy controls in an eye-closed resting condition.
5. As above.
6. Confounding factors: medication and age.
7. Age was statistically adjusted, and this study did not include anyone who took medications that would affect the EEG.
8. Yes QEEG was used.
9. No exposure.
10. Met assumptions for parametric statistics. No corrections for multiple comparisons were made.

**Zinn et al. 2018**

1. Participants matched by age, sex, educational level and ethnicity. Confirmed with statistics.
2. Participants were current, past-study and wait- listed patients Stanford University CFS clinic. Location radius of HCs were not provided.
3. Patients with CFS were identified by the Fukuda et al. (1994) criteria within 6 months prior to participation through clinical diagnosis. Inclusion and exclusion criteria—absence of known neurological disorders, seizure activity, brain trauma, psychiatric, or physical diseases different than CFS—were assessed during telephone screening. Participants with neurological or psychiatric conditions were also excluded based on medications they reported as currently taking due to medication effects in the EEG (e.g. anxiolytics, anti-depressants, sedatives, etc.) HCs were included if they had no exclusionary medical disorders and no abnormal physical functioning.
4. No exposure. Study investigated cortical hypoactivation during resting EEG suggests CNS pathology in patients with ME/CFS and HCs.
5. As above.
6. Confounding factors: medication and exclusionary criteria.
7. Participants that had any of the mentioned exclusionary criteria were controlled for via exclusion. Acceptable medication consumption was ceased 24-hours prior to the EEG.
8. Yes, EEG was used. Behavioural aspects were also measured using two different measures: Multidimensional Fatigue Inventory (MFI-20) (Smets, Garssen, Bonke, & De Haes, 1995) and the Fatigue Severity Scale (FSS). This has excellent reliability and validity (Cronbach’s alpha: 0.84)
9. No exposure.
10. Yes, normalisation was performed. To control for Type 1 error, significant voxels were identified using a statistical non-parametric mapping (SnPM) procedure. Corrected P-values were calculated using supra-threshold cluster-size tests.
