# Supplementary material for: A systematic review of neurological impairments in myalgic encephalomyelitis/ chronic fatigue syndrome using neuroimaging techniques
Source: PLoS One. 2020 Apr 30;15(4):e0232475. doi: 10.1371/journal.pone.0232475 (PMC7192498; doi:10.1371/journal.pone.0232475)
Supplement: Attachment — Submitted filename: Response to Reviewers.docx [file pone.0232475.s009.docx]

Miss Rebekah Maksoud

National Centre for Neuroimmunology and Emerging Diseases

Griffith University

Queensland, Australia

Email: [ncned@griffith.edu.au](mailto:ncned@griffith.edu.au)

15^th^ April 2020

Marcello Moccia

Academic Editor

PLOS ONE

Dear Marcello Moccia,

**Re: Manuscript number** (PONE-D-19-34471); “A Systematic Review of neurological impairments in myalgic encephalomyelitis/ chronic fatigue syndrome using neuroimaging techniques.”. **Authors:** Rebekah Maksoud, Stanley du Preez, Natalie Eaton-Fitch, Kiran Thapaliya, Leighton Barnden, Hélène Cabanas, Donald Staines, Sonya Marshall-Gradisnik

The authors would like to thank you and all the reviewers for providing their time in reading this systematic review and responding with constructive feedback. We greatly appreciate the reviewer comments and have modified text to reflect these minor reviewer comments.

We have now re-submitted a clean modified manuscript reflecting reviewer 1 and reviewer 3 comments and suggestions as well as a track changes manuscript. The authors wish to highlight to the Editor, the suggestion by Reviewer 1 for the inclusion of a particular journal paper, Kimura *et al, was not included due to the authors followed the* standardised and internationally accepted **PRISMA and** **Cochrane review guidelines** (<https://training.cochrane.org/handbook/current/chapter-04#section-4-4>). Reviewer 1 again suggested the inclusion of this paper; however, this suggested paper did not meet our international and standardised PRISMA and Cochrane review protocols. Thus, inclusion of this paper would introduce bias from self-selection of papers. However, the authors have modified the discussion to include descriptions of the paper’s findings.

Please refer to the attached page for our responses to minor comments provided by each reviewer.

We would also like to extend our thanks to the editorial team for their timely return of this manuscript.

Sincerely,

*Rebekah Maksoud*

**Reviewer #1:**

**Reviewer #1, comment 1:** The authors wrote to comment 1: "Kimura et al, was excluded during the abstract/ title screening process as it did not contain any of the listed key words. "

This is not wrong, however, because this paper seems to be within the scope of this manuscript with regards to its content, the reviewer is still not completely convinced why this paper is excluded from this review. Please explain this point.

**Authors Response:** **Considered and modified in the manuscript.** Our systematic review utilised standardised and internationally accepted PRISMA and Cochrane review guidelines (<https://training.cochrane.org/handbook/current/chapter-04#section-4-4>). This approach limits the introduction of bias from self-selection of papers. The inclusion of Kimura *et al* although within the scope of our review would be introducing a paper outside the set international guidelines and stringent inclusion criteria and may be a source of potential selection bias. The discussion has been modified to include descriptions of the paper’s findings (line 494).

**Reviewer #3:**

**Reviewer #3, comment 1:** Reviewer #3: Fukuda criteria is way too broad, and such a review should have addressed some articles with more restrictive criteria. There is a strong lack of consensus among researchers and clinicians on it's reliability as far as specificity is concerned.

**Authors Response:** **Considered and modified in the manuscript.** A statement in the discussion was provided regarding the limitation of including the Fukuda criteria in our search strategy: **“**Inclusion of the Fukuda criteria was a limitation of our search strategy, however, due to this criteria being still abundantly used in research and clinical practice, limiting our search to not include papers that relied on the Fukuda criteria would drastically reduce the availability of appropriate papers in this field.” (line 423)
